# Full-waveform inversion reveals diverse origins of lower mantle positive wave speed anomalies

**DOI:** 10.1038/s41598-024-77399-2

**Published:** 2024-11-04

**Authors:** Thomas L. A. Schouten, Lars Gebraad, Sebastian Noe, Anna J. P. Gülcher, Solvi Thrastarson, Dirk-Philip van Herwaarden, Andreas Fichtner

**Affiliations:** 1 Structural Geology and Tectonics, Geological Institute, Department of Earth and Planetary Sciences, ETH Zurich, Sonneggstrasse 5, 8092 Zurich, Switzerland; 2 Seismology and Wave Physics, Institute of Geophysics, Department of Earth and Planetary Sciences, ETH Zurich, Sonneggstrasse 5, 8092 Zurich, Switzerland; 3 Planetary Interiors and Geophysics Division, Jet Propulsion Laboratory, California Institute of Technology, Pasadena, CA USA; 4 Seismological Laboratory, Division of Geological and Planetary Sciences, California Institute of Technology, Pasadena, CA USA

**Keywords:** Seismic tomography, Plate tectonics, Mantle heterogeneity, Geodynamics, Geology, Seismology, Tectonics

## Abstract

Determining Earth’s structure is paramount to unravel its interior dynamics. Seismic tomography reveals positive wave speed anomalies throughout the mantle that spatially correlate with the expected locations of subducted slabs. This correlation has been widely applied in plate reconstructions and geodynamic modelling. However, global travel-time tomography typically incorporates only a limited number of easily identifiable body wave phases and is therefore strongly dependent on the source-receiver geometry. Here, we show how global full-waveform inversion is less sensitive to source-receiver geometry and reveals numerous previously undetected positive wave speed anomalies in the lower mantle. Many of these previously undetected anomalies are situated below major oceans and continental interiors, with no geologic record of subduction, such as beneath the western Pacific Ocean. Moreover, we find no statistically significant correlation positive anomalies as imaged using full-waveform inversion and past subduction. These findings suggest more diverse origins for these anomalies in Earth’s lower mantle, unlocking full-waveform inversion as an indispensable tool for mantle exploration.

## Introduction

Solid-state convection of the rocky, 2,890-km deep mantle has shaped the evolution of Earth’s interior and surface over billions of years. Uncovering Earth’s internal structure and the distribution of thermal and compositional heterogeneity, however, remains a scientific challenge that requires cross-disciplinary efforts. Seismic tomography represents the primary method to image the Earth’s interior, leveraging anomalies in wave speed stemming from heterogeneities in its thermal and chemical structure. Classical travel-time tomography reveals numerous regions of large, positive seismic wave speed anomalies throughout Earth’s mantle^1–3^ that are robust features across models^4^ (Suppl. text S1). Because seismic wave speed is a non-unique expression of a combination of material parameters^5^, however, interpreting the nature of any imaged anomaly is inherently challenging. Commonly, positive wave speed anomalies in the mantle are attributed to the presence of a cold^6^ and/or chemical anomaly (e.g. Fe-, Mg- or Si- enrichment)^7^ (Suppl. text S2). There is abundant geochemical and geophysical evidence suggesting that Earth’s mantle hosts chemical heterogeneity at a variety of scales^8–14^, which is corroborated by geodynamic simulations^15–19^. However, the positioning of positive seismic wave speed anomalies in the lower mantle directly below - or proximal to - locations of modern and ancient subduction zones have prompted their interpretation as (remnants of) cold subducted plates, or ”slabs”^6,20,21^ (Suppl. text S2). The advent of quantitative global plate reconstructions^22^ revealed a statistically significant correlation ($p \le 0.01$) between these positive anomalies and past subduction^23^. A corollary to this interpretation is that the seismically imaged mantle structure reflects dominantly thermal as opposed to thermochemical heterogeneity^24–26^.

**Figure 1:**
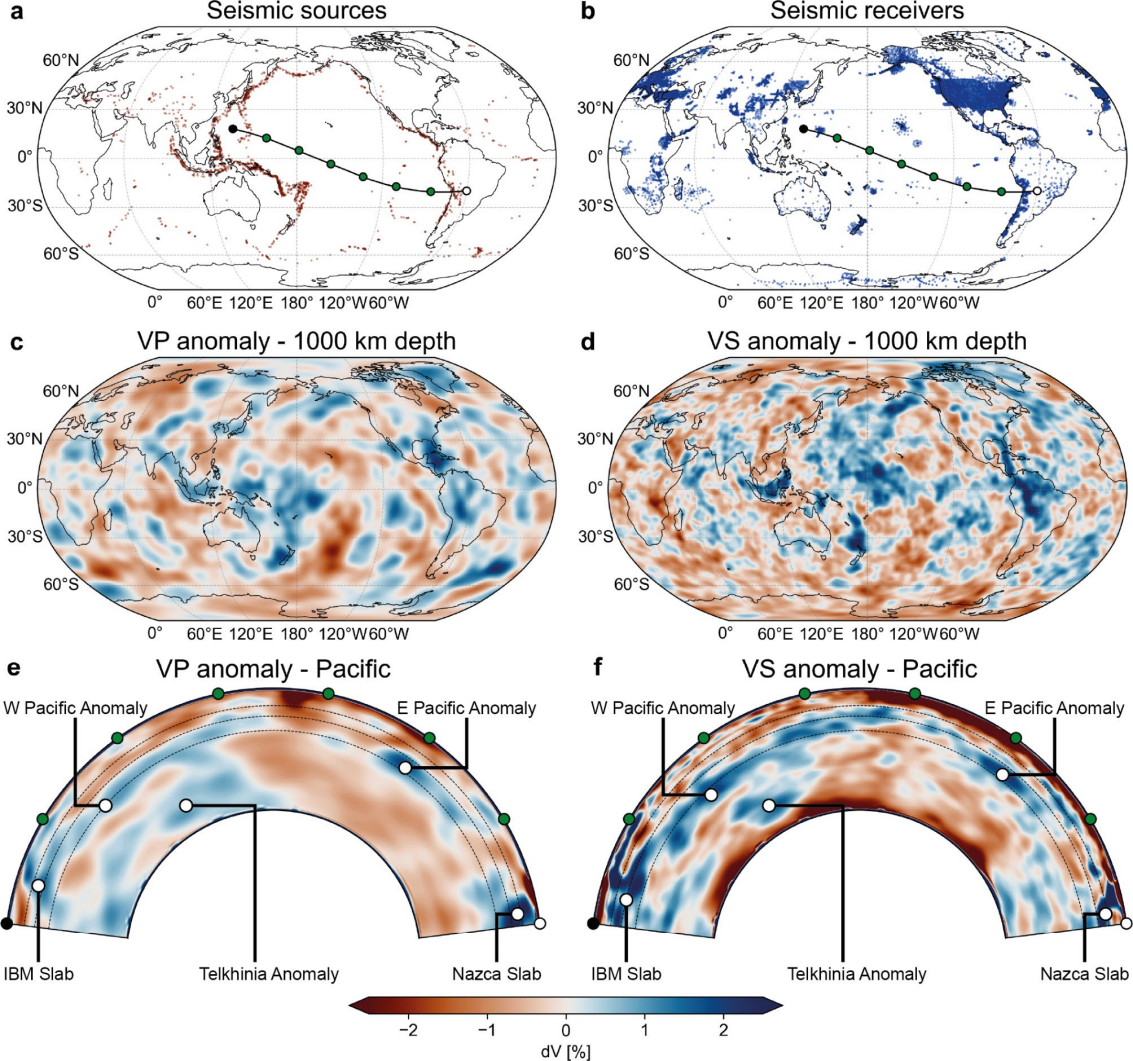
Global distribution of seismic stations, receiver locations, and seismic wave speed anomalies used to construct the FWI model REVEAL. Seismic stations and receiver locations are presented in (**a,b**), respectively; seismic wave speed anomalies are shown at a depth of 1000 km in (**c,d**) and a cross-section across the Pacific in (**e,f**). The trace of the cross sections is shown in (**a**). VP: P-wave speed; VS: S-wave speed. Note that the resolvable amplitude of wave speed anomalies is higher for S-waves than for P-waves. Of particular interest is the presence of large positive wave speed anomalies below the Pacific, Atlantic, and Indian oceans, as seen in (**e,f**). IBM: Izu-Bonin-Marianas. Telkhinia Anomaly from^27,29^.

The assumption that most, if not all, lower mantle positive wave speed anomalies represent cold slabs led to a detailed correlation of each anomaly with a geologic record of subduction^27^. and has been widely applied to reconstruct ancient plates and subduction zones^28–31^ (Suppl. text S3), determine the absolute motions of tectonic plates^32,33^, infer mantle density structure^6,34^ along with its convective dynamics^33,35–37^ and resulting dynamic topography^38,39^ (Suppl. text S2), and even to estimate atmospheric CO$_2$ levels in the geologic past^40^. Yet, travel-time tomographic models are typically constructed by inverting the travel times of only a few easily identifiable body wave phases, mostly direct waves (Suppl. text S1). As these phases only have resolving power following the geometry of their predicted rays^3^ or volumetric sensitivity kernels^41^, the tomographic resolution of these models is highly sensitive to the non-uniform global distribution of sources and receivers (Fig. 1a, b)^42^. A key question for the current understanding of mantle structure and dynamics is thus whether the spatial bias of travel-time tomography affects the correlation between positive anomalies in the (lower) mantle and reconstructed subduction zones^23^ and by extension, the assumption that (almost) all positive wave speed anomalies represent dominantly thermal heterogeneities introduced to the mantle by subduction^6,20,21,27,32,34–36,40^ (Suppl. text S2).

**Figure 2:**
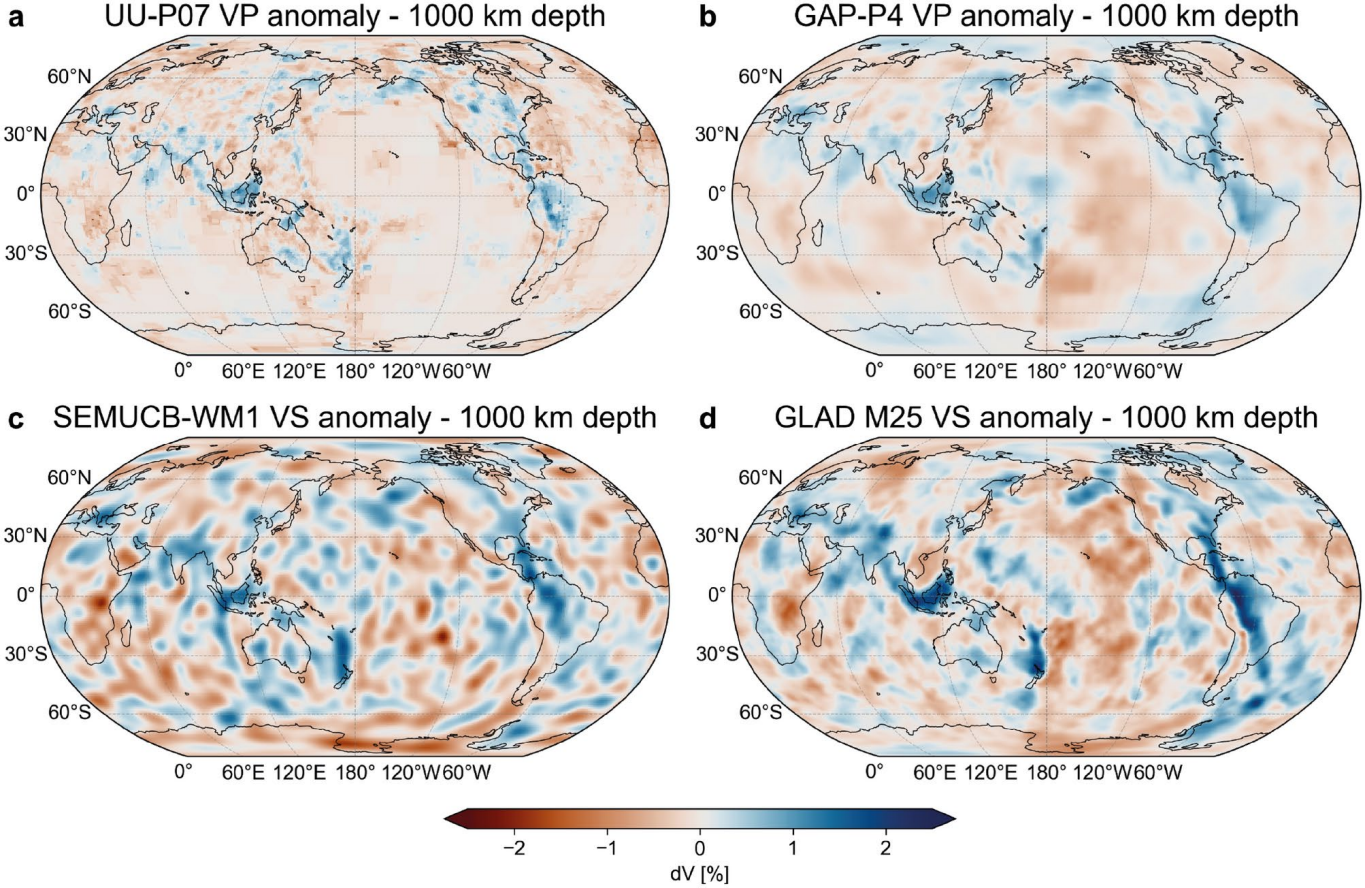
Comparison of other tomographic models at 1000 km depth. (**a**) UU-P07 (P-wave speed, ray)^46^. (**b**) GAP-P4 (P-wave speed, finite-frequency)^47^. (**c**) SEMUCB-WM1 (S-wave speed, hybrid-waveform inversion)^45^. (**d**) GLAD-M25 (S-wave speed, full-waveform inversion)^44^.

**Figure 3:**
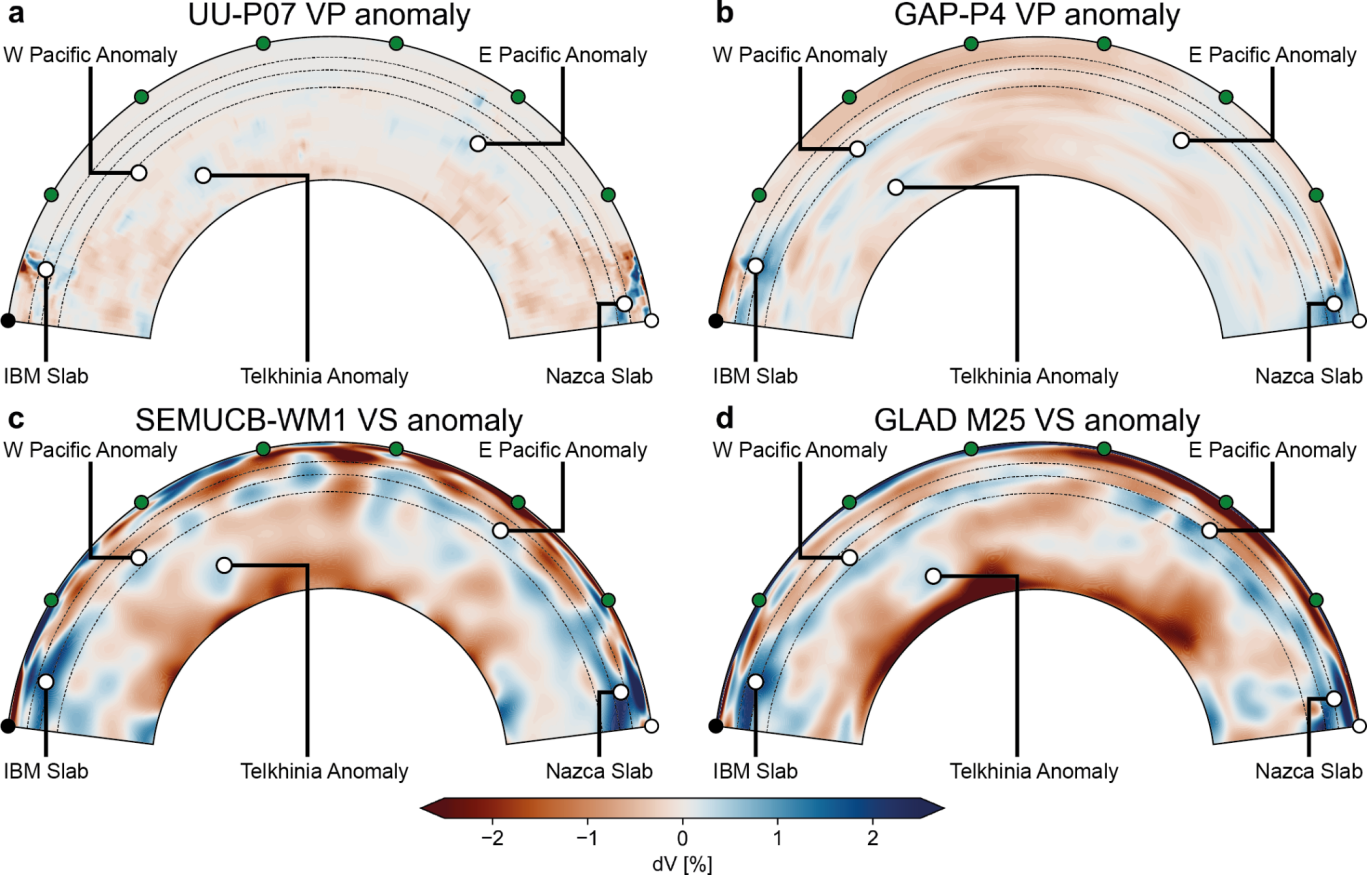
Comparison of cross sections through the Pacific ocean of wave speed anomalies in other tomographic models. (**a**) UU-P07^46^ (P-wave speed, ray). (**b**) GAP-P4^47,48^ (P-wave speed, finite-frequency). (**c**) SEMUCB-WM1^45^ (S-wave speed, hybrid-waveform inversion). (**d**) GLAD-M25^44^ (S-wave speed, full-waveform inversion). The trace of the cross sections is given in Fig. 1a in the main article.

Here, we analyse Earth’s seismic wave speed structure constructed using full-waveform inversion (FWI), which has greater volumetric sensitivity to Earth’s mantle than travel-time tomography. Global FWI reveals a much more complex, heterogeneous mantle seismic wave speed structure than traditionally imaged^43–45^. In particular, it reveals numerous large, positive wave speed anomalies in the mid- and lower mantle, even below major oceans and continental interiors with low seismic activity and/or limited station coverage, and importantly, no geologic record of subduction (Figs. 1, 2, 3). We find that one of the most pronounced newly-detected fast wave speed anomalies lies beneath the western Pacific Ocean between 900-1200 km depth. Through wavefield simulations, we show that FWI is sensitive to anomalies in this region, highlighting how this method can recover mantle structure beneath regions without seismic sources and/or receivers. Furthermore, we show that there is no statistically significant correlation between positive wave speed anomalies in the lower mantle with the reconstructed locations of former subduction zones for a global FWI. These results suggest that not all positive wave speed anomalies in the lower mantle are thermal anomalies resulting from slabs that subducted in the last 200 Ma, and that they therefore do not represent a reliable proxy to reconstruct past subduction nor to directly constrain the thermal and density structure of the mantle. Finally, we show how our observations are compatible with alternative, previously proposed origins for positive wave speed anomalies in the lower mantle^8–19^, illustrating the potential of FWI for future mantle exploration.

## Imaging Earth’s mantle

Tomography provides information on the three-dimensional distribution of physical properties inside an otherwise inaccessible medium that affect wave propagation. For the seismic waves that traverse Earth’s interior, these properties include elastic, anelastic, and anisotropic parameters, as well as material density^49^. Seismic tomographic results are typically presented as anomalies relative to a reference model such as the Preliminary Reference Earth Model (PREM)^50^. Because seismic wave speed is dependent on a range of material parameters, these anomalies are by definition non-unique expressions of heterogeneity in the medium.

Global travel-time tomography represents a groundbreaking technique that illuminated mantle structure for the first time^1–3^ and with remarkable consistency^4^ (Suppl. text S1). However, classical ray tomography relies on the selection and correct identification of a few teleseismic body wave phases from seismograms to constrain bulk seismic properties along the ray paths between sources and receivers. Commonly, only direct P- or S-phases are selected, rendering this approach very sensitive to source-receiver geometry. Although this is true for any tomographic inverse problem, the approximation of body waves as travelling along infinitely thin ray paths leaves particularly large gaps in volumetric sensitivity^3^, which can only be partially alleviated using explicit volumetric sensitivity kernels in finite-frequency tomography^41^. Another approach is to include travel times of other, reflected and refracted body wave phases. However, these constitute only a small part of travel-time datasets as these phases are harder to detect from seismograms (Suppl. text S1). On Earth, most sources and receivers are located along major (convergent) plate boundaries that are concentrated along the Tethyan belt, which stretches from Iberia to SE Asia, and around the margins of the Pacific Ocean (Fig. 1a, b). By contrast, stable plate interiors such as much of the African continent, Antarctica, North Asia, and major oceans are mostly devoid of both sources and receivers. This results in a much higher tomographic resolution in the mantle beneath plate boundaries compared to plate interiors^42^. Moreover, as the seismic wave speed increases with depth due to mineral phase transitions and increasing density from compression, the majority of the ray paths and volumetric sensitivity kernels beneath plate interiors cluster in the lowermost mantle, rendering a large portion of the lower mantle ($\sim$660-2000 km depth range) poorly resolved (Fig. S1)^3,32^. Alternative methods such as normal-mode tomography, which is only sensitive to long-wavelength structures, and surface-wave tomography also lack resolution in this depth range^51,52^.

Full-waveform Inversion (FWI) attempts to overcome these limitations by fitting entire seismograms, rather than only a selection of body wave phases. This includes the reflected and refracted body wave phases that effectively increase the volumetric sensitivity of the inversion to cover the entire mantle. Moreover, it incorporates more information on mantle seismic structure per earthquake than travel-time tomography without the need for prior identification of individual phases. FWI was conceptualised in the early 1980s^53–55^ and further developed in the early 2000s when computing power allowed for its practical three-dimensional use^56–58^. However, the large computational cost of simulating wave propagation for the entire globe precluded its application to whole-mantle tomography. To mitigate this, global FWI models rely on efficient usage of seismic data through GPU accelerated numerical simulations^59^, dynamic mini-batching^60^ or wavefield-adaptive meshing^61^. Resulting models such as GLAD-M25^44^ and REVEAL^43^, have much improved resolution compared to ray or finite-frequency tomography models.

## Distribution and resolution of positive wave speed anomalies from FWI

All FWI models^43–45^ reveal significantly more seismic wave speed heterogeneity in both lateral distribution and amplitude than travel-time tomographic models (Figs. 1, 2, 3 & S2). In particular, we find large, positive wave speed anomalies in regions of the mantle where travel-time tomography has a low resolution. The largest anomaly in the FWI model REVEAL is located directly below the western Pacific Ocean, a region that is practically devoid of seismic sources and/or receivers (Fig. 1). Here, a large, tabular, positive wave speed anomaly is apparent between 900-1200 km depth, albeit with variable lateral and radial extent for P- and S-wave speed anomalies (Fig. 1). Furthermore, a set of smaller, vertically oriented anomalies at similar depths is located west of South America (Fig. 1). The amplitude, depth range, and morphology of these newly-detected anomalies are similar to that of upper mantle anomalies interpreted as actively subducting slabs imaged beneath the Izu-Bonin-Marianas and Nazca subduction zones, as well as that of lower mantle anomalies disconnected from the surface commonly inferred to represent detached slab remnants^27,28,30,31^. These newly-detected anomalies are also apparent in other waveform-based models such as SEMUCB-WM1^45^ and GLAD-M25 but practically absent in all travel-time tomography models^4^ (Figs. 2, 3 & S2, Suppl. text S1). This is a direct consequence of the source-receiver geometry: because sources and receivers surrounding the Pacific are separated by $\sim$90$^{\circ }$, the P- and S-phases that constitute the bulk of the data used in travel-time models traverse this region through the lowermost mantle rather than the mid mantle (Fig. S1).

**Figure 4:**
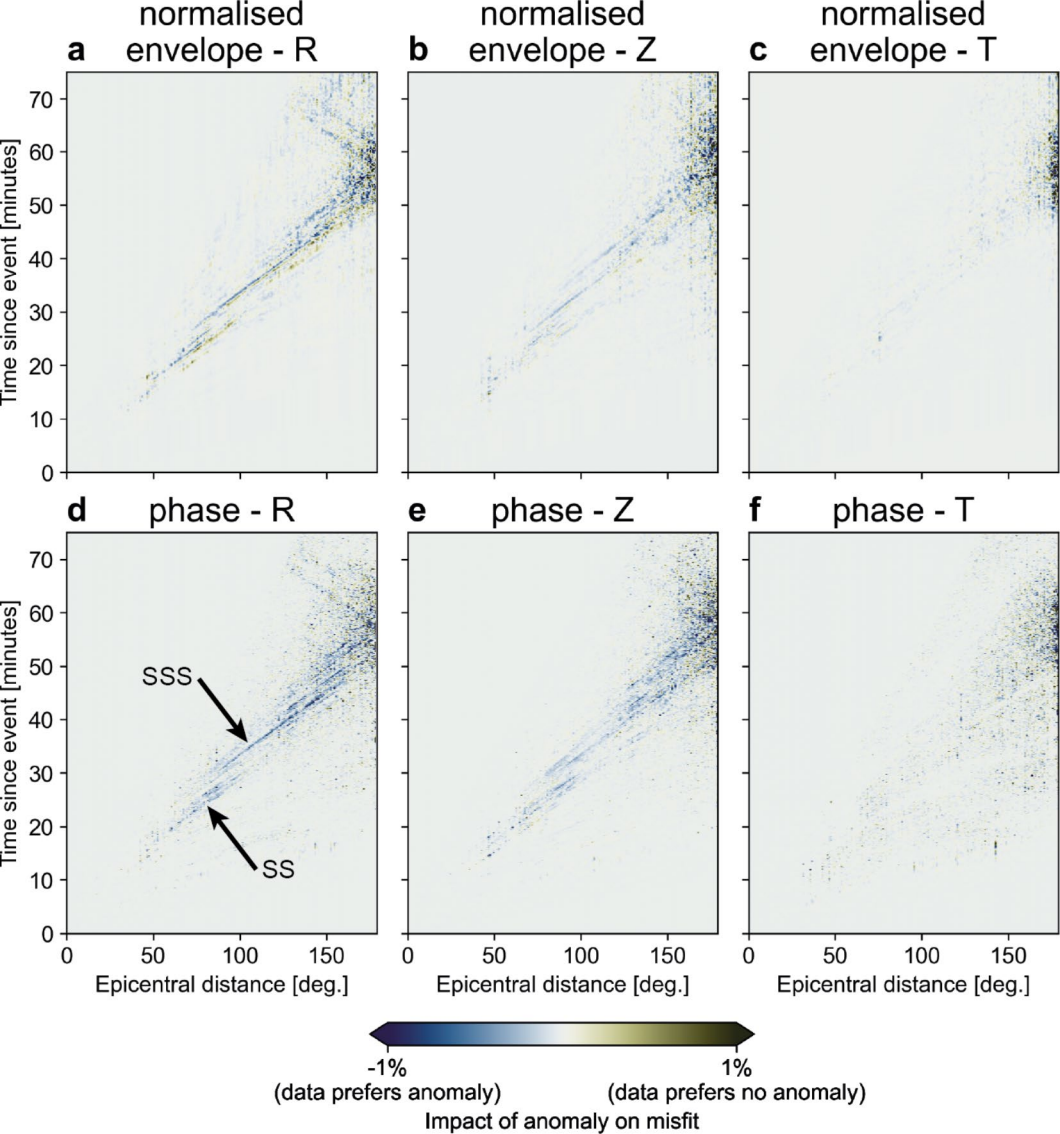
Difference between computed seismograms for REVEAL with and without the seismic anomaly beneath the Pacific ocean. (**a**) Normalised envelope misfit for the radial components. (**b**) Normalised envelope misfit for the vertical components. (**c**) Normalised envelope misfit for the vertical components. (**d**) Wave phase misfit for the radial components. (**e**) Wave phase misfit for the vertical components. (**f**) Wave phase misfit for the transverse components. Note the clear contribution of the SS and SSS wave phases to the sensitivity of REVEAL to velocity anomalies beneath the western Pacific (**d**).

Although FWI theoretically alleviates this problem by exploiting full seismograms, its volumetric sensitivity remains dependent on the source-receiver configuration. All three published waveform-based models - REVEAL, GLAD-M25, and SEMUCB-WM1 - have been validated by accurately predicting three-component seismograms for a wide range of source-receiver combinations not included in their inversions.^43–45^. This independent testing of FWI is crucial in interpreting anomalies, ensuring they are not erroneous results of model adjustments due to noise in the data. For additional verification of the sensitivity of global FWI to structure below the western Pacific, we simulated the full wavefield and computed synthetic seismograms for a representative selection of earthquakes whose wavefields traverse this region. For this analysis, we use REVEAL, the most recently published FWI model, as it is the only global-scale model capable of resolving structures typically visible only in high-resolution, regional-scale tomography models^43^. Simulations were conducted for both the complete wave speed structure of the model and a modified version with the western Pacific anomaly removed to evaluate the impact of this feature on waveform misfits. (Fig. S3, Materials and Methods). The results show that global FWI is sensitive to wave speed anomalies below the western Pacific and that especially the radial and vertical components of the synthetic seismograms are better fit with the data with the anomaly included in REVEAL (Fig. 4). We identify the travel times of SS and SSS-wave phases, not universally used in travel-time mantle tomography (Tables S1-S3, Suppl. text S1), to be sensitive to wave speed anomalies below the western Pacific, amongst several other phases that are not easily identified (Fig. 4); the large number of phases sensitive to this anomaly illustrates how picking only a few easily identifiable ones omits crucial information on mantle structure from the inversion. The generally higher amplitude of those anomalies in FWI that correspond to known anomalies in travel-time tomographic models likely results from the remaining source-receiver bias in FWI: the Fresnel zone width of volumetric sensitivity kernels, which is dependent on the source-receiver distance, means that wave speed anomalies imaged in regions that do not host any sources and/or receivers are averaged over a larger volume compared to regions that do, damping the resolvable amplitudes. In conclusion, the results from global FWI^43–45^ are robust and have superior resolution compared to travel-time tomography in the mid and lower mantle of the Earth.

## Challenging the correlation of lower mantle positive seismic wave speed anomalies and past subduction

Although P- and S-wave speeds are dependent on a range of material properties, positive wave speed anomalies are typically assumed to represent dominantly thermal anomalies introduced to the mantle through subduction in most applications of mantle tomography^6,27–30,32,34–36,38–40^. While some of the positive anomalies in the lower mantle are still connected to the surface, most of these appear as isolated features interpreted as detached slab remnants. Slab detachment is expected, for example, by tearing of oceanic lithosphere from more buoyant continental lithosphere during continental collision^62,63^, by the densification of oceanic crust as it undergoes the basalt-to-eclogite phase change at high pressures^64^, and/or by local weakening through grain-size reduction in cold slabs that enter the lower mantle^7,65^.

**Figure 5:**
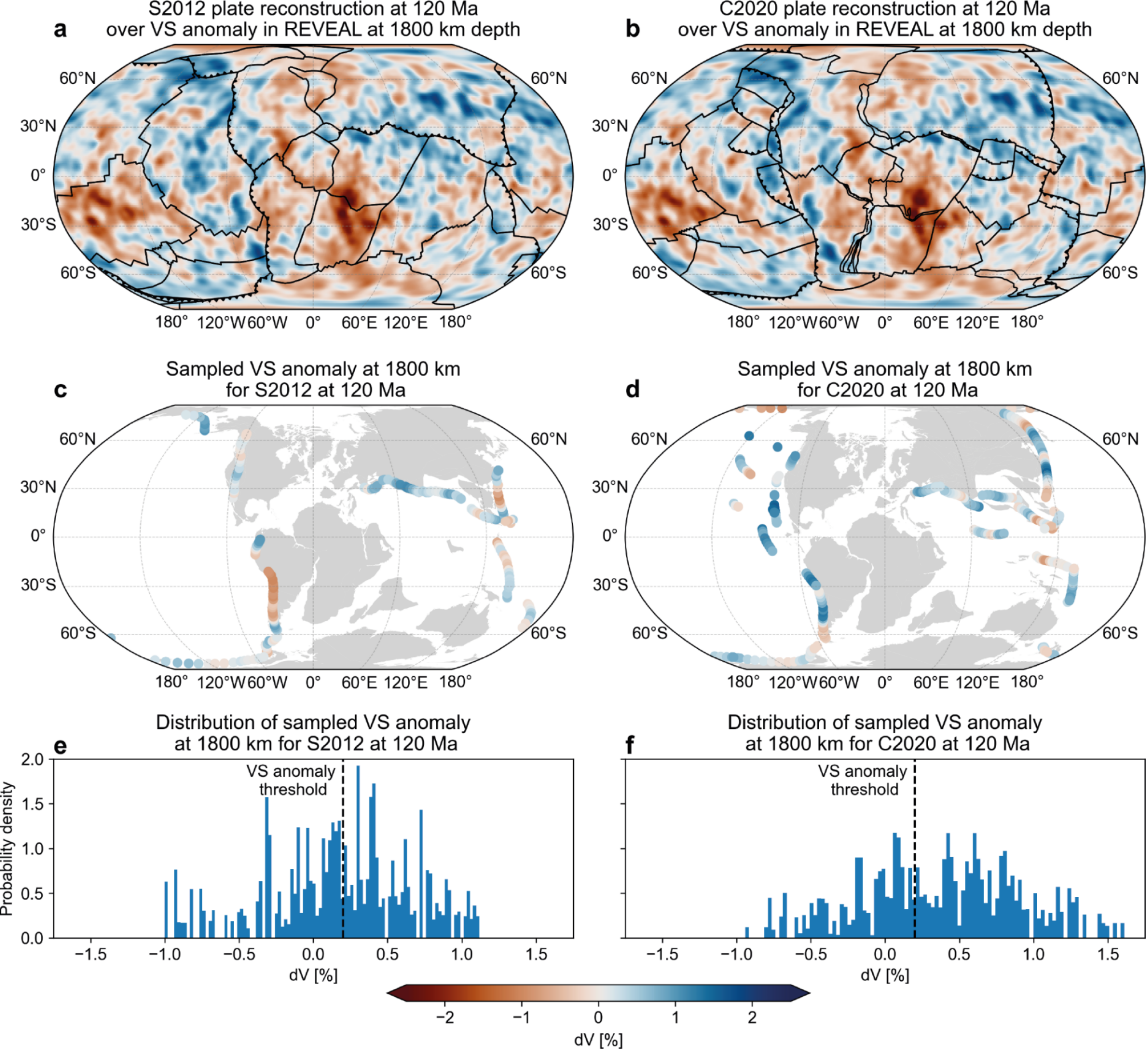
Workflow for the correlation of positive wave speed anomalies with reconstructed subduction zones. Top panels (**a,b**) show continents and plate boundaries in the S2012 and C2020 reconstructions at 120 Ma plotted over the VS wave speed anomaly in REVEAL at 1800 km depth. Middle panels (**c,d**) show wave speed anomaly at 1800 km sampled at reconstructed subduction zones for both reconstructions. Panels (**e,f**) show the distribution of sampled wave speed anomaly weighted by the length of each subduction zone segment for both reconstructions.

Domeier et al.^23^ corroborated this assumption by showing that there is a linear, time-depth progressive correlation between positive S-wave speed anomalies in the 600–2300 km depth range from several travel-time tomography models and the location of subduction zones in the last 130 Ma as reconstructed by Seton et al.^22^ (S2012 , Fig. 5a, c), that is significant at the 1% significance level. Their findings imply that slabs sink essentially vertically at a rate of 1.1–1.9 cm/a and that their morphology is representative of the former location and geometry of the subduction zone where they entered the mantle, which is widely applied to develop new plate reconstructions^28–32^.

With continuous improvements in tomographic resolution, facilitated by e.g. finite-frequency and waveform-based inversions, the number of detected positive anomalies in Earth’s mantle has steadily risen. Consequently, there has been a parallel increase in the number and cumulative length of subduction zones with each newly published reconstruction (Suppl. text S3), leading to complex subduction geometries such as in the ”tomotectonic” reconstruction of Clennett et al.^28^ (C2020, Fig. 5b, d, Suppl. text S2). Despite this increasing complexity however, the overall organisation of Earth’s plate tectonic system is similar across plate reconstructions: since the assembly of Pangaea ($\sim$300 Ma), subduction zones have been concentrated in the northern margin of the Tethyan realm and around the Pacific-Panthalassa realm^22,28,30,66^ (Fig. S4, Suppl. text S2). Interestingly, these areas roughly coincide with the locations of present-day plate boundaries as well as seismic sources and receivers (Figs. 1a, b, S4, Suppl. text S3), meaning that the expected locations of the now-subducted slabs correspond to the regions with the highest resolution in travel-time tomography. In contrast, FWI reveals the presence of many more positive wave speed anomalies distributed throughout the mantle (Figs. 1, 2, 3, S4). In fact, the western Pacific anomaly (Fig. 1) is located directly below the absolute plate motion path of the oldest part of the Pacific Plate since its formation around 180 Ma (Fig. 5a, b)^22,66^. Moreover, the eastern Pacific anomalies (Fig. 1) are located beneath the East Pacific Rise, a long-lived spreading centre (Fig, 5a, b)^22,28^. These observations question the previously established correlation between positive wave speed anomalies and subduction zones^23^, and its applications^27,28,32,37^.

**Figure 6:**
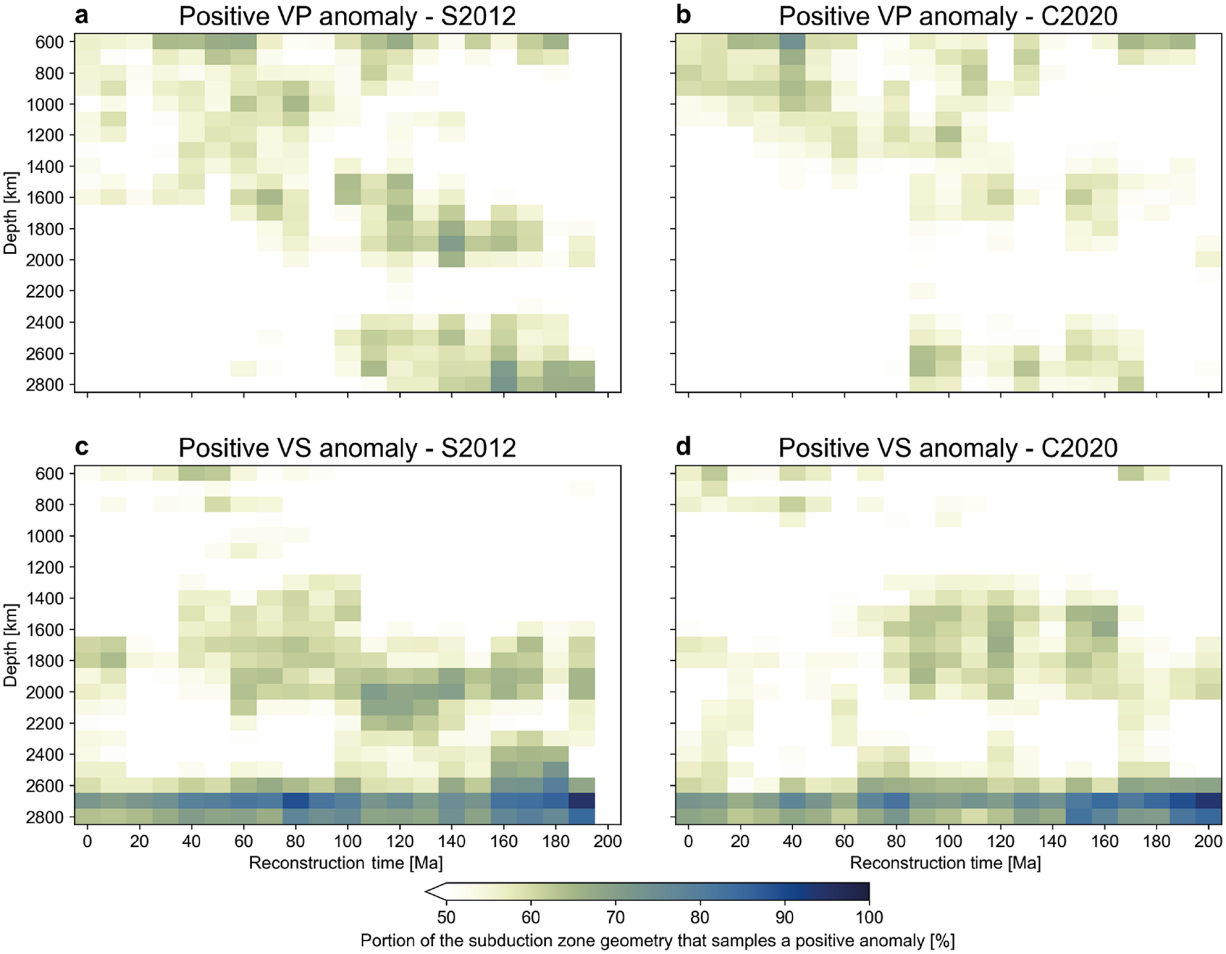
Correlation between subduction zones and positive seismic wave speed anomalies. 2D histograms of time-depth comparison of the portion (in percent) of the subduction geometries that sample a positive VP or VS anomaly for two reconstructions: S2012^22^ in (**a,c**) and C2020^28^ in (**b,d**).

To assess this correlation, we repeat a modified version of the experiment by Domeier et al.^23^ (Materials and Methods). We again select REVEAL for this experiment, but given that the overall distribution of positive anomalies is similar to that in GLAD-M25 and SEMUCB-WM1, these results are representative for any global FWI model. We calculate the fraction of the total length of the subduction zones in the S2012 and C2020 reconstructions that sample a positive anomaly, defined as $\ge 0.1~dVP$ and $\ge 0.2~dVS$^27,28,30^ for the time-depth combinations of 0-200 Ma and 600-2800 km in 10 Ma and 100 km intervals, respectively (Fig. 6, Materials and Methods). Only up to $\sim$60-70% of any subduction geometry actually samples a positive anomaly, contradicting the assumption of a direct one-to-one correspondence. Nevertheless, we find a weak time-depth progressive correlation of the fraction of P-wave speed anomalies sampled by either reconstruction that roughly corresponds to the sinking rate of 1.1-1.9 cm/a found by Domeier et al.^23^ (Fig. 6). In contrast, we find no clear time-depth progression for S-wave speed anomalies, with all reconstructed subduction geometries sampling more than 50% positive anomalies in the 1400-2200 km depth interval. Interestingly, the ”tomotectonic” C2020 reconstruction, which aims to improve the S2012 reconstruction by inferring additional subduction zones from tomography, generally samples a lower fraction of positive anomalies (Fig. 6, Suppl. text S3).

**Figure 7:**
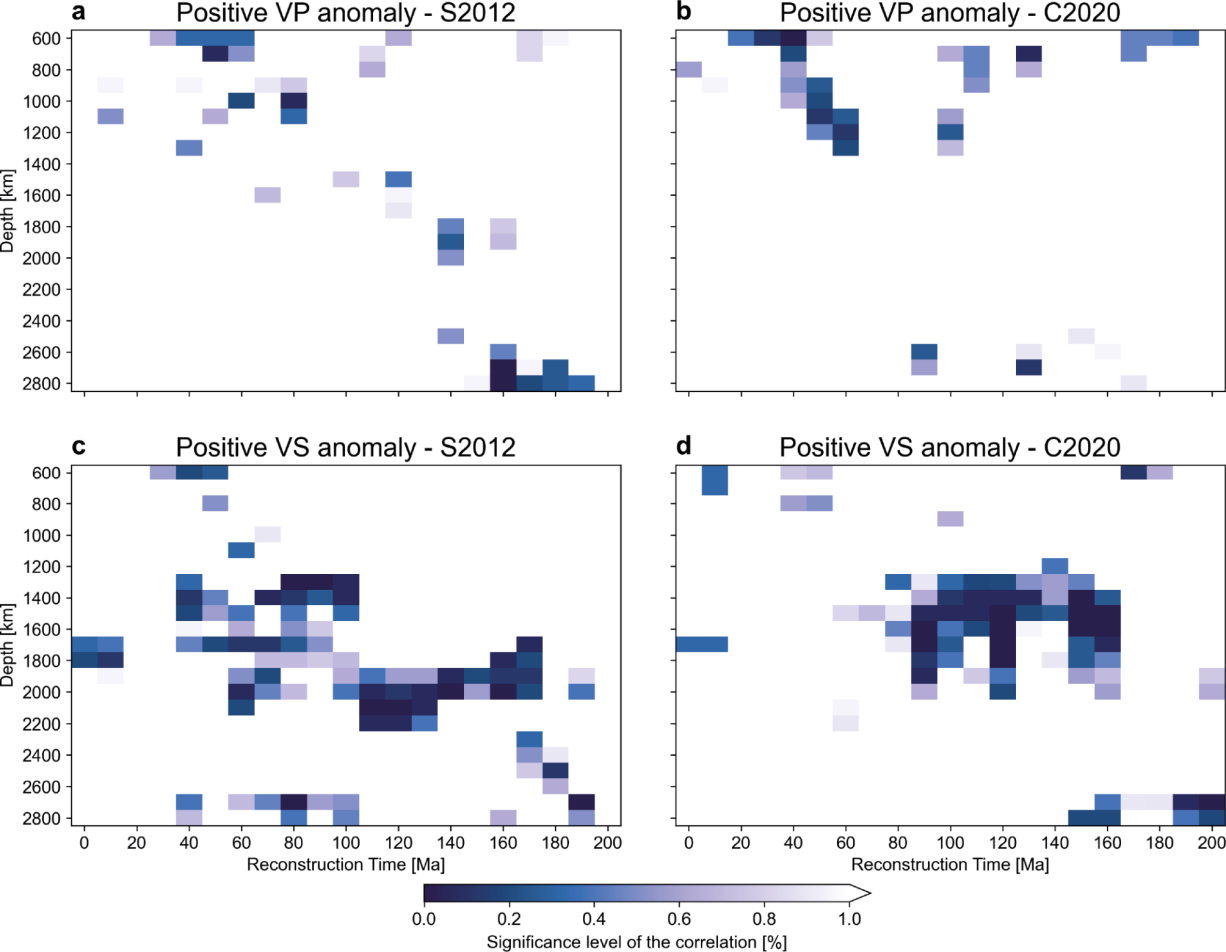
Significance of the correlation between subduction zones and positive seismic wave speed anomalies. Time-depth comparisons for which the null hypothesis of no correlation between positive anomalies and past subduction can be rejected at the 1% significance level (Main text, “Materials and methods”).

To test the significance of these correlations, we repeat the experiment of Domeier et al.^23^: for each time-depth combination, we compare the fraction of positive anomalies sampled at the reconstructed subduction geometry to those sampled at $10^5$ randomly rotated instances of that geometry under the null hypothesis of no correlation between positive anomalies and subduction (Materials and Methods). The results show that the weak time-depth progressive correlation between positive P-wave speed anomalies and past subduction is rejected at the 1% significance level (Fig. 7a, b). We do find several time-depth correlations for S-wave speed anomalies for the 1400-2200 km depth range (Fig. 7c, d). Yet, these show no time-depth progression and are compatible with slab sinking rates ranging from $\sim$1 cm/a to a physically implausible $\sim$16 cm/a (Fig. 7c, d). Intriguingly, the ”tomotectonic” C2020 reconstruction yields correlations that are statistically less significant than those in the S2012 reconstruction (compare Fig. 6a, c with Fig. 6b, d, respectively), which we suspect is primarily a result of the difference in absolute reference frame (see Suppl. text S3 for discussion).

Given that the overall distribution of positive anomalies in all FWI models^43–45^ is similar (Figs. 1 and 2), these observations suggest that the correlation obtained by Domeier et al.^23^ likely stems from a spatial bias in travel-time tomography. This directly challenges the commonly presumed one-to-one correspondence of positive wave speed anomalies to subducted slabs; although a significant portion of the positive anomalies imaged by FWI likely represent slabs, their distribution and morphologies may not serve as reliable bases to reconstruct plates and subduction zones^28–30,40^. Moreover, our analysis raises questions about the statistical robustness of previously inferred slab sinking rates^23,27^, suggesting these should be employed with caution to constrain absolute plate motions^32,33^ or calibrate mantle convection models^36,37^.

## Reconciling mantle heterogeneity with FWI tomography

There is a diverse range of potential explanations for the detection of positive wave speed anomalies in Earth’s (lower) mantle other than the presence of subducted slabs. For example, the base of the lithosphere may delaminate and sink into the mantle as a result of gravitational instability. Such instability may arise due to the presence of dense, eclogitic, lower crustal root^67^, old, thick, and cold lithosphere^68,69^, and/or small-scale convection^70,71^. Furthermore, chemical heterogeneities may emerge from the segregation of the high-density oceanic crust (basalt) from subducted and/or delaminated oceanic lithosphere, particularly in the mantle transition zone^8,72,73^. Mantle rocks enriched in basalt generally have higher seismic wave speeds than harzburgite-enriched (i.e. depleted) rocks in the upper mantle due to the formation of dense, Si-rich phases such as garnet and clinopyroxene. In the lower mantle, these Si-rich phases transform into high-pressure phases like bridgmanite, post-perovskite, and stishovite, which also have increased seismic velocities^7,74^. These basalt-enriched streaks may remain unmixed, generating a poorly-mixed ”marble cake” mantle structure^75,76^. In fact, the prolonged survival of recycled oceanic crust in the mantle transition zone and/or the lower mantle of the Earth is a robust geochemical prediction^10,11^ that is supported by geodynamic modelling^15,18,19^. Such compositional anomalies may be laterally displaced throughout the mantle by lateral mantle flow^73,77,78^ and/or by encountering strong compositional contrasts that deflect downgoing material^16,17,79–81^.

Particularly for the mid mantle beneath the Pacific Ocean, evidence from REVEAL (Fig. 1) suggests the existence of chemical and thermal anomalies unrelated to subduction, which is endorsed by other studies of the region. For example, the oldest lithosphere of the Pacific Plate is thinner than expected from lithospheric half-space cooling models^82^, and it has been proposed that the base of the lithosphere has been removed through small-scale convection^83^; the western Pacific anomaly may thus represent the now-delaminated base of the plate. Alternative theories regarding the mid-mantle structure beneath the Pacific involve a variety of phenomena: a regional mantle discontinuity at $\sim$1000 km depth caused by a deflected lower mantle plume^9,14^; the presence of a Si-enriched geochemical reservoir at this depth range^12,15^; or basalt-harzburgite segregation in the proximity of the Hawaiian Mantle Plume^13^. This diverse range of hypotheses, along with our analysis of REVEAL (Figs. 1 and 4), collectively suggest that wave speed anomalies detected beneath the Pacific reflect (thermo-)chemical instead of dominantly thermal heterogeneities related to subduction. This questions the practice of scaling wave speed anomalies to temperature and density, along with the convective dynamics predicted by such scaling (Suppl. text S2)^34,35,38,39^.

In conclusion, the prevailing assumption that positive wave speed anomalies in the lower mantle solely represent the thermal signature of subducted slabs is incomplete. Rather, this assumption reflects the spatial resolution of the few easily identifiable body wave phases typically included in travel-time tomography. Our study reveals widespread and large-scale mantle heterogeneity, consistent with previous predictions from geochemical data and geodynamic modeling. These predictions suggest a variety of sources for positive wave speed anomalies, including cold, dense delaminated lithospheric mantle, as well as domains with dense, Si-rich phases, possibly due to basalt-harzburgite segregation or ancient mantle heterogeneities. Importantly, our research underscores the critical role of Full Waveform Inversion as an indispensable tool in mantle exploration and encourages future research to further delineate Earth’s complex mantle structure.

## Materials and methods

### Wavefield modelling

To demonstrate the resolving power of full waveforms in the mid mantle beneath the Pacific Ocean, we employ numerical simulations for two models of Earth’s wave speed structure using SALVUS^84^. We generate a copy of REVEAL where we remove the western Pacific anomaly by attenuating it to the background average wave speed for all seismic wave speed components (Fig. S3), thereby moving it away from the optimal model solution back to the starting model. We select 40 earthquakes in the magnitude range 6.0–6.5 with a deliberate emphasis on hypocenters around the Pacific Ocean for which we generate synthetic seismograms from the wavefield propagating through the two Earth models (Fig. 1). Note that while the selected sources are mostly located around the Pacific, the seismograms are evaluated for all available stations around the globe (Figs. 1a, b, S3a, b). These synthetic seismograms are evaluated in the period range of 35–120 s. The impact of the anomaly on the misfit $\chi$ is gauged by comparing the difference of the computed seismograms and the observations for both models, i.e. $1 - \frac{\chi (\text {no-anomaly})}{\chi (\text {REVEAL})}$. We identify seismic phases using the epicentral distance and time since the event.

### Statistical correlation

We repeat a modified version experiment of Domeier et al.^23^ to statistically correlate positive wave speed anomalies in the lower mantle to reconstructed subduction zones. We extract the reconstructed subduction zones for the last 200 Ma from two end-member global plate reconstructions^22,28^ as a series of points that represent individual subduction zone segments using GPlately^85,86^. We follow the filtering and downsampling approach of Domeier et al.^23^ and remove any subduction zones with 80% transcurrent motion and convergence rates lower than 0.1$^{\circ }$/Ma ($\approx$1.11 cm/a), as well as downsampling REVEAL to a 1$^{\circ }$ grid to filter out small-scale variations. We then sample the magnitude of the anomaly at the subduction zones for all seismic wave speed components. Domeier et al.^23^ compared the mean of the velocity anomaly magnitude sampled at the true subduction geometry to $10^5$ instances of randomly rotated subduction geometries under the null hypothesis that the true mean is not larger than any randomly sampled mean. The problem with this approach however is that the amplitude of an imaged velocity anomaly is not just dependent on the heterogeneity in the medium but also on the non-uniform global tomographic resolution (Main text). We instead calculate the fraction of the total subduction zone length that samples a positive anomaly. We define a positive velocity anomaly as any part of the mantle with a velocity anomaly above 0.1% and 0.2% for P- and S-wave speed anomalies, respectively. These thresholds are comparable to those used to identify ”slabs” in the mantle in the tomotectonic or slab unfolding workflows^27,28,30^. Because the location of slabs may be slightly offset relative to past subduction zones due to bending, lateral motion and/or thickening of the slab, we sample not only at the subduction zone itself but also 200 km (the typical arc-trench distance) and 400 km inboard of the overriding plate; these modifications do not affect the results (compare Figs. S5–S7). We also doubled the threshold amplitude of a positive anomaly to 0.2% and 0.4% for P- and S-wave speed anomalies, respectively, which correspond to the highest threshold values used to identify ”slabs”^27,28,30^; For these thresholds, practically none of the reconstructed subduction geometries sample more than 50% anomalies (Fig. S7).

We test the statistical significance of these results by repeating the experiment of Domeier et al.^23^: we compare the fraction of the subduction geometry that samples a positive anomaly to that of 10$^5$ randomly rotated instances of the same subduction geometry under the null hypothesis that there is no correlation between positive wave speed anomalies and the reconstructed locations of former subduction zones. The subduction geometry is rotated using a uniform random rotation matrix^87^. Whereas the rotation axes of this matrix are uniformly distributed on the unit sphere, the rotation angles follow the probability density function $P_{\alpha } (\alpha ) = \frac{1}{\pi } sin^2 \frac{\alpha }{\pi }$, with a range of rotation angles $-\pi \le \alpha \le \pi$. This effectively rotates the subduction geometry such that it uniformly covers the globe in all possible orientations after all iterations. The results are expressed as the statistical significance of the null hypothesis, i.e. a percentage of 0% means that the fraction of the true subduction geometry that samples a positive anomaly is higher than that of 100% of the randomly oriented instances. Following Domeier et al.^23^, we select 1% as the cutoff for a statistically significant correlation.

## Supplementary Information

Supplementary Information.Philippine Sea Plate and Australasian oceans and orogens

## Data Availability

The global distribution of sources and receivers, tomographic models (netCDF4 format), stacked waveforms from the wavefield modelling (.h5 format), reconstruction files (GPlates-compatible format), 2D grids of the time-depth correlations (.csv format), and Python scripts required for the full analysis and figures presented in the main text and supplementary information are included in the supplementary data folder, which is available on Zenodo (10.5281/zenodo.13235438). SALVUS is available from Mondaic (https://mondaic.com). Parameters to run the wavefield experiments are available upon request.
