# Supplementary material for: Full-waveform inversion reveals diverse origins of lower mantle positive wave speed anomalies
Source: Sci Rep. 2024 Nov 4;14:26708. doi: 10.1038/s41598-024-77399-2 (PMC11535529; doi:10.1038/s41598-024-77399-2)
Supplement: Supplementary file 1 — Supplementary Information.Philippine Sea Plate and Australasian oceans and orogens [file 41598_2024_77399_MOESM1_ESM.pdf]

Supplementary material for: Full-waveform inversion reveals diverse origins of lower mantle positive wave speed anomalies

Thomas L.A. Schouten[1,*,+], Lars Gebraad[2,+], Sebastian Noe[2,+], Anna J.P. Gülcher[3,4], Solvi Thrastarson[2], Dirk-Philip van Herwaarden[2], Andreas Fichtner[2]

October 25, 2024

[1]Structural Geology and Tectonics, Geological Institute, Department of Earth and Planetary Sciences, ETH Zurich, Sonneggstrasse 5, 8092, Zurich, Switzerland
[2]Seismology and Wave Physics, Institute of Geophysics, Department of Earth and Planetary Sciences, ETH Zurich, Sonneggstrasse 5, 8092, Zurich, Switzerland
[3]Seismological Laboratory, Division of Geological and Planetary Sciences, California Institute of Technology, Pasadena, California, USA
[4]Planetary Interiors and Geophysics Division, Jet Propulsion Laboratory, California Institute of Technology, Pasadena, California, USA

[*] To whom correspondence should be addressed; E-mail: tschouten@eaps.ethz.ch.
[+] These authors contributed equally to this work

S1 Comparison of tomographic models

Most travel-time mantle seismic tomographic models are constructed from a one-dimensional starting model such as PREM (Dziewonski and Anderson, 1981) or IASP91 (Kennett and Engdahl, 1991) by inverting the travel times of P- or S- wave phases along ray paths (Amaru, 2007; Simmons et al., 2010). More recent tomographic models have extended the input data to other, refracted and/or reflected body wave phases (Houser et al., 2008; Li et al., 2008; Simmons et al., 2012; Burdick et al., 2017; Hosseini et al., 2020) or have included surface waves and/or normal modes (Grand, 2002; Ritsema et al., 2011; Auer et al., 2014). Because these other wave phases are harder to identify on seismograms, they constitute only a small part of the datasets used in these models. For instance, while S40RTS (Ritsema et al., 2011) incorporates a wide range of phases including SSS, SKS, SKKS, as well as (multiple) core and (multiple) surface reflections, S and SS phases still constitute 42.3% and 27.2%, respectively, of the total travel time dataset. Finally, recent models account for the volumetric sensitivity of each of these phases in finite-frequency tomography (Yomogida, 1992) rather than using the ray approximation (Obayashi et al., 2013; Li et al., 2008; Montelli et al., 2004, 2006; Burdick et al., 2017; Hosseini et al., 2020). Despite these differences, the overall distribution of wave speed anomalies in the lower mantle is generally similar for all travel-time models (Becker and Boschi, 2002; Trampert and Spetzler, 2006; Shephard et al., 2017; Rahimzadeh Bajgiran et al., 2023) (Figs 2, 2 & S2).

Another class of seismic tomographic models is constructed by inverting waveforms (French and Romanowicz, 2014; Lei et al., 2020; Thrastarson et al., 2024). Given the key advantages of these waveform-based models over ray or finite-frequency tomography (Main text), we compare the distribution of wave speed anomalies in the lower mantle for two travel-time and two waveform-based tomographic models (Table S1), as well as vote maps (Shephard et al., 2017) for six P- and six S-wave speed anomaly models (Tables S2 & S3, respectively).

One key observation is that the recoverable amplitudes of P-wave speed anomalies are up to a factor 4 times larger in REVEAL than in UU-P07 (Amaru, 2007) and GAP-P4 (Obayashi et al., 2013). Furthermore, the distributions of wave speed anomalies are different: whereas P-wave and S-wave tomographic models resolve major positive anomalies in the vicinity of sources and receivers (Main text and Figs. 1-3, S2), waveform-based models image additional positive anomalies below the Atlantic, Indian, and Pacific Oceans (Main text and Figs. 1-3). These anomalies correspond to known zones of relatively low resolution in travel-time tomographic models (van der Meer et al., 2012). We identify both the western and eastern Pacific anomalies (Main text) in the two waveform-based models, corroborating our analysis of REVEAL.

Of course, waveform-based models come with some degree of uncertainty. Methods to quantify uncertainty in FWI usually involve perturbing the optimised model solution (e.g. at points (Fichtner and Trampert, 2011) or in a checker-board pattern (van Herwaarden et al., 2023)), and recomputing waveforms to see if i) the changes significantly affect the objective function and ii) the reconstructed image corresponds to the artificially introduced perturbation. However, these methods require substantial computational resources and storage capacity to be conclusive, and even then may not lead to the intended results (Lévêque et al., 1993). As we are solely interested in showing that any kind of resolving power exists in the mid-mantle beneath the Pacific with FWI, we designed a hypothesis test specifically for the anomaly. Hereby, we imitate the established uncertainty quantification methods by perturbing the original model REVEAL (Thrastarson et al., 2024) and moving it away from the optimal model solution, however, we perform this step in a targeted and meaningful manner by reverting the anomaly back to the starting model. The results in Fig. 4 indicate that the changes in computed waveforms are, in fact, significant for the objective function as they are perceived above the noise level and show the expected shift in arrival time for seismic phases. We therefore conclude that we have sensitivity in the area of interest while refraining from quantifying the resolution explicitly due to computational constraints and because the result are unlikely to impact the findings of this study.

Table S1: **Models directly analysed in our comparisons (see Figs. 1-3).**

Model name	Original work	Inversion method	Used data
UU-P07	Amaru (2007)	ray approximation	Body waves (P)
GAP-P4	Obayashi et al. (2013)	finite-frequency	Body waves (P)
SEMUCB-WM1	French and Romanowicz (2014)	hybrid full-waveform inversion	Waveforms
GLAD-M25	Lei et al. (2020)	full-waveform inversion	Waveforms
REVEAL	Thrastarson et al. (2024)	full-waveform inversion	Waveforms

Table S2: **Ray and finite-frequency tomographic models included in the VP vote map.** Models selected after Shephard et al. (2017). Vote map at 1000 km depth is presented in Fig. S2a.

Model name	Original work	Inversion method	Used data
GyPSuM-P	Simmons et al. (2010)	ray approximation	Body waves (P)
HMSL-P06	Houser et al. (2008)	ray approximation	Body waves (P, PP)
LLNL_G3Dv3	Simmons et al. (2012)	ray approximation	Body waves (P, Pn)
MITP_2016	Li et al. (2008), Burdick et al. (2017)	finite-frequency	Body waves (incl. P, PP, Pdiff)
PRI-P05	Montelli et al. (2004, 2006)	finite-frequency	Body waves (P, PP)
DETOX-P3	Hosseini et al. (2020)	finite-frequency	Body waves (P, PP, Pdiff)

Table S3: **Ray and finite-frequency tomographic models included in the VS vote map.** Models selected after Shephard et al. (2017). Vote map at 1000 km depth is presented in Fig. S2b.

Model name	Original work	Inversion method	Used data
GyPSuM-S	Simmons et al. (2010)	ray approximation	Body waves (S)
HMSL-S06	Houser et al. (2008)	ray approximation	Body waves (S, SS)
PRI-S05	Montelli et al. (2006)	finite-frequency	Body waves (S, SS-S, ScS-S)
S40RTS	Ritsema et al. (2011)	ray approximation	Surface waves, body waves (incl. S, SS, SSS), normal modes
SAVANI	Auer et al. (2014)	ray approximation	Surface waves, body waves (incl. S, SS, SSS)
TX2011	Grand (2002)	ray approximation	Surface waves, body waves (incl. S, SS, SSS), normal modes

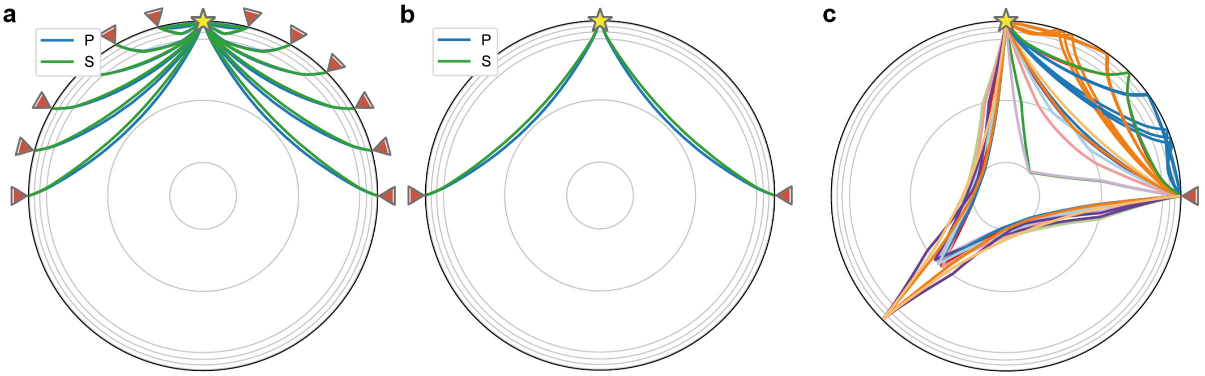

Figure S1: **Sensitivity of mantle structure to different wave phases.** Panel a) shows the ray paths of P- and S-phases with a station spacing of 10°, illustrative of the typical tomographic resolution along a convergent plate boundary. Panel b) shows the ray paths of P- and S-phases for a single source-receiver pair separated by 90° illustrating how direct waves through regions with low seismic activity and/or stations pass through the lowermost mantle. Panel c) shows the ray paths of all phases at 90°, illustrating how using the full seismogram enhances the volumetric resolution of the inversion. Note that the use of the ray approximation is for illustrative purposes only since FWI explicitly accounts for the volumetric sensitivity of all phases. Figure generated using TauP Crotwell et al. (1999) in ObsPy Krischer et al. (2015) for a PREM wave speed structure Dziewonski and Anderson (1981).

S2 Interpretation of positive wave speed anomalies

Interpreting mantle heterogeneity from tomography presents a significant challenge, mostly because seismic velocities depend on a range of material properties (Main text) (Deschamps and Trampert, 2003; Trampert et al., 2001). Furthermore, the magnitude of an imaged velocity anomaly is also influenced by the non-uniform global tomographic resolution (Main text, Materials and Methods). Interpreting seismic wave speed from a purely thermal perspective suggests that positive anomalies are associated with material that is colder (and therefore denser) than the surrounding mantle, and that negative anomalies are linked to areas with hotter (and consequently more buoyant) material (Schuberth et al., 2009; Davies et al., 2015). However, chemical variations that result in buoyant material may also be expressed as positive wave speed anomalies, and vice-versa (McNamara and Zhong, 2005; Deschamps et al., 2015).

Since most anomalies imaged by classical travel-time tomography can be attributed to subduction (Main text, Suppl. text S3), most geodynamic interpretations of mantle tomography consider wave speed anomalies as thermal in nature. While subduction, partially driven by the high density of (meta-)basalt, introduces chemical heterogeneity into the mantle, the positive wave speed anomalies interpreted to be subducted plates are mostly considered as dominantly thermal anomalies. A common practice is to directly scale the magnitude of the relative wave speed anomaly to the magnitude of the relative thermal density anomaly (Becker and O'Connell, 2001; Steinberger and Calderwood, 2006; Müller et al., 2008; Faccenna and Becker, 2010; Ghosh et al., 2010; Becker and Faccenna, 2011; Steinberger and Torsvik, 2012; Straume et al., 2024; Conrad et al., 2004). The resulting mantle density distributions are used to predict a velocity field that is used for (back)advection of the material, deducing present-day mantle flow patterns (Becker and O'Connell, 2001; Conrad et al., 2004; Steinberger and Calderwood, 2006; Faccenna and Becker, 2010; Ghosh et al., 2010; Becker and Faccenna, 2011) as well as mantle flow in the geologic past (Müller et al., 2008; Steinberger and Torsvik, 2012; Straume et al., 2024). Moreover, these derived flow patterns are correlated to regional dynamic topography anomalies in the present-day (Conrad et al., 2004) or in the geologic past (Müller et al., 2008; Straume et al., 2024). Overall, despite the non-uniqueness of seismic wave speed, the thermal interpretation of wave speed anomalies is deeply rooted in geophysical and geodynamic research.

S3 Comparison of plate reconstructions

Reconstructing relative and absolute plate motions

Tectonic reconstructions are a powerful tool to study any aspect of the plate tectonic cycle (Seton et al., 2023). In particular, it represents the only deep-time constraint on mantle convection Gerya (2019); Seton et al. (2023). The cornerstone of any tectonic reconstruction since the Jurassic is the global plate circuit: marine magnetic anomalies and fracture zones in oceanic basins record the relative divergent plate motion between major plates (McKenzie and Parker, 1967; Müller et al., 1997; Seton et al., 2012). Combining these allows the construction of a circuit that provides the relative motions between all plates connected to it.

Whereas the relative motions of these tectonic plates are relatively well known, their absolute motions (i.e. relative to the mantle) can only be constrained by a set of assumptions on the nature of plate motions and/or mantle convection. For instance, fixed- or moving-hotspot reference frames assume a quasi-static position of mantle plumes below hotspots such as the Canaries, Iceland, Réunion, Hawaii, and Galápagos (Doubrovine et al., 2012; O'Neill et al., 2005; Torsvik et al., 2010). The S2012 reconstruction uses the fixed hotspot reference frame of O'Neill et al. (2005) until 100 Ma and assumes Africa is stationary with respect to the lower mantle between 100-200 Ma. Slab reference frames minimise the cumulative distance between positive anomalies and the reconstructed subduction zones to which they are inferred to correspond (van der Meer et al., 2010; Domeier et al., 2016). Finally, geodynamic reference frames are optimised to fit both hotspot locations as well as a set of *a priori* assumptions on the maximum rates of trench migration and net lithospheric rotation (Tetley et al., 2019). The C2020 reconstruction uses the geodynamic reference frame of Tetley et al. (2019) for 0-250 Ma. However, all these reference frames produce very low rates ($<0.15°$/Ma) of net lithospheric rotation relative to the mantle, with similar results obtained from geodynamic modelling (Zhong, 2001; Becker, 2006; Conrad and Behn, 2010). This is important because it implies that the distribution of seismic sources and receivers correlates with the expected locations of subducted slabs, if they sink vertically (Fig. S4).

While the plate circuit can constrain the amount of convergence between plates, it does not provide evidence of the location of the subduction zone(s) that separated these. Furthermore, absolute reference frames may constrain the absolute motion of a plate, but cannot directly constrain the location where a plate subducted into the mantle. Reconstructing these subduction zones requires careful restoration of the modern orogenic architecture using structural, sedimentological and palaeomagnetic data from their rock record (van Hinsbergen and Schouten, 2021).

Reconstructing subduction zones

The destructive nature of subduction removes much of the evidence of both downgoing and overriding plates from the rock record. To overcome this problem, recent tectonic reconstructions increasingly leverage the supposed correlation between positive wave speed anomalies and subducted plates. In the "tomotectonic" workflow, subduction zones are drawn directly above slabs inferred from positive anomalies, with the time-depth correlation estimated using an *a priori* constant sinking rate (Clennett et al., 2020; Mohammadzaheri et al., 2021; Sigloch and Mihalynuk, 2013, 2017). In the "slab unfolding" workflow, subducted plates are reconstructed through surface projection of the volume of positive anomalies interpreted as slabs, accounting for the thickening of these slabs as they descends into the mantle (Chen et al., 2019; Fuston and Wu, 2020; Wu et al., 2016; Wu and Suppe, 2018; Wu et al., 2022). Both work-

flows assume that the boundaries of a ”slab” can be defined by a threshold velocity anomaly of around $\geq$0.0-0.2%.

Neither of these workflows, however, critically evaluate why a particular anomaly necessarily represents a subducted slab and why must represent the associated volcanic arc on the overriding plate. A concrete example where critical evaluation of the geologic record allows for the identification of a subduction-related versus a non-subduction-related anomaly is in the reconstruction of the Alisitos and Guerrero terranes by C2020. Here, the authors interpret the elongated, E-W oriented ”ALI” anomaly as representing subducted Farallon lithosphere. Following their workflow, they reconstruct an E-W oriented subduction zone between 140 and 115 Ma where the Farallon Plate subducts below their “Alisitos Plate”, so named because they interpreted the elongated Alisitos Terrane as the volcanic arc that formed above this subduction zone. Based on the absence of volcanic rocks younger than 115 Ma (Johnson et al., 1999), they interpret subduction ended at this time, and the Alisitos Plate was captured by the Farallon Plate. Given that this terrane is now oriented NNW-SSE, this requires the arc to have been subject to a 90° vertical axis rotation, which they reconstruct between 110 and 103 Ma during accretion of the Alisitos Terrane along the western active margin of the Guerrero Terrane, which they reconstruct on a separate, Guerrero Plate. Interestingly however, there is no surface geologic (i.e. structural geologic or palaeomagnetic) data supporting such vertical axis rotation, however, in the literature presented by the authors (Wetmore et al., 2014). In fact, the Alisitos and Guerrero are considered laterally equivalent terranes because they share a stratigraphy consisting of Lower Cretaceous volcanic and volcaniclastic rocks, interpreted as a volcanic arc, overlying Triassic-Jurassic a diverse range of metasedimentary rocks with a detrital zircon age distribution similar to that of North America, interpreted as an accretionary prism (Boschman et al., 2018, and references therein). The simplest interpretation of these geologic constraints is thus to reconstruct the Alisitos and Guerrero terranes on a single plate rather than two separate plates, which suggests that the ALI anomaly of C2020 may not represent a subducted slab.

Global plate motions since the Mesozoic

Since the amalgamation of the supercontinent Pangaea at $\sim$350 Ma, Earth’s plate tectonic system consisted of two distinct realms: in the triangular Tethyan Realm, plates moved from Gondwana in the South to Laurasia in the North, accommodated by predominantly E-W striking spreading ridges and subduction zones (Stampfli and Borel, 2002; Seton et al., 2012). In the Panthalassa Realm, plates moved away from the oceanic centre towards the margins of the supercontinent of Pangaea (Engebretson et al., 1985; Larson and Chase, 1972; Seton et al., 2012; Torsvik et al., 2019). While consensus exists over the large-scale tectonic organisation, plate reconstructions tend to deviate in the distribution and number of subduction zones due to differences in the used input data or reconstruction approach (Seton et al., 2023; Boschman et al., 2014; van Hinsbergen et al., 2020). Here we briefly analyse where the S2012, C2020, and alternative plate reconstructions diverge and whether this would affect our results from the statistical correlation (Main text).

The Atlantic Realm has been almost exclusively characterised by oceanic spreading since $\sim$200 Ma (Seton et al., 2012; Matthews et al., 2016), with the Caribbean and Scotia subduction zones only invading the Atlantic Realm in the last $\sim$30 Ma (Boschman et al., 2014; Eagles and Jokat, 2014; Müller et al., 2019; Nerlich et al., 2013; van de Lagemaat et al., 2021). C2020 implements several regional reconstructions (Boschman et al., 2014; Eagles and Jokat, 2014; Müller et al., 2019; Nerlich et al., 2013) that are in terms of subduction geometry, not significantly different from the more recent reconstruction by van de Lagemaat et al. (2021). The

African and North- and South American continental interiors have not been affected by any major tectonic events in the last ∼350 Ma (Heine et al., 2013; Seton et al., 2012).

The rock record of the Tethyan Orogenic Belt provides evidence of several intraoceanic subduction zones that may have been active at the location of the present-day Indian Ocean in the last ∼200 Ma (Barber and Crow, 2009; Beck et al., 1996; Martin et al., 2020; Khan et al., 2007; Westerweel et al., 2019; Zhu et al., 2016). However, these are reconstructed proximal to the southern active margin of Eurasia or in the vicinity of the Junction Region where the Tethyan and Pacific-Panthalassa Realm interacted (Advokaat and van Hinsbergen, 2023; Gaina et al., 2015; Gibbons et al., 2015; Hall, 2012; van de Lagemaat and van Hinsbergen, 2023; Wu et al., 2016; Zahirovic et al., 2014). Although neither the S2012 nor the C2020 reconstructions implement all of the subduction zones proposed by these alternative reconstructions, the overall subduction geometry for the Tethyan Realm remains largely similar. The Junction Region between the Tethyan and Pacific-Panthalassa realms is where C2020 implements the regional reconstruction of Zahirovic et al. (2014), resulting in a much more complex subduction geometry than in the original S2012 reconstruction.

The Pacific-Panthalassa Realm has been dominated by radial oceanic spreading, with subduction concentrated at the surrounding circum-Pacific orogens. Although there is evidence in the rock record of these orogens for small intra-Panthalassa subduction zones in the last ∼250 Ma (Bazhenov et al., 2001; Konstantinovskaia, 2001; Johnston, 2001; Nokleberg et al., 2001), these are reconstructed proximal to the margins of the Pacific-Panthalassa realm (Clennett et al., 2020; Domeier et al., 2017; Fuston and Wu, 2020; Lin et al., 2022; Vaes et al., 2019; Wu et al., 2016, 2022; Zahirovic et al., 2014; van de Lagemaat and van Hinsbergen, 2023). The most extensive and complex of these proposed subduction geometries was implemented in the C2020 reconstruction in the Eastern Pacific Basin relative to the S2012 reconstruction. For the NW Pacific the C2020 reconstruction implemented a simplified scenario based on higher resolution regional reconstructions (Domeier et al., 2017; Lin et al., 2022; Vaes et al., 2019; Wu et al., 2022); despite this simplification, these reconstructions do not significantly diverge from the C2020 reconstruction in terms of number, geometry, and cumulative length of their subduction zones. Finally, the C2020 incorporates a regional tomotectonic reconstruction of the Arctic Region (Shephard et al., 2013) whose subduction geometry does not significantly diverge from the S2012 reconstruction. In conclusion, the S2012 and C2020 can be considered end-member plate reconstructions in terms of the number and cumulative length of subduction zones; the number and cumulative length of subduction zones in alternative reconstructions typically lie within the spectrum delineated by these two end-members. Despite their regional differences, the global subduction geometry remains broadly similar across all reconstructions.

Differences in correlations of positive anomalies with reconstructed subduction zones

Our results show that, compared the S2012 reconstruction, the subduction geometry of C2020 samples a smaller fraction of positive anomalies across most time-depth combinations for both P- and S-wave anomalies. Additionally, the correlation for C2020 is generally weaker, with more time-depth combinations having significance levels below 1%. A significant correlation is only observed with positive S-wave anomalies in the 80-170 Ma time window and the 1400-2000 km depth range, without a clear time-depth progression. This counterintuitive result may be explained by two factors. First, the significant correlation observed in the 80-170 Ma time range in C2020 directly corresponds to the existence of the complex Mesozoic subduction geometry in the eastern Pacific Basin in this reconstruction (Fig. 5b). During this period, the length of this eastern Pacific subduction geometry accounts for ∼20-30% of the total global

subduction geometry length (Fig. S8). The 1400-2000 km depth range aligns with the depth of those positive wave speed anomalies that were interpreted as subducted slabs by Clennett et al. (2020) and form the foundation for this complex geometry. Therefore, this significant correlation is a logical consequence of the ”tomotectonic” approach. Second, the absence of significant correlations outside this time-depth range may be attributed to differences in the absolute reference frames used in the reconstructions. While the S2012 reconstruction relies on a fixed hotspot reference frame (O'Neill et al., 2005), the C2020 reconstruction employs the geodynamic reference frame of Tetley et al. (2019), which was optimised to simultaneously fit hotspot tracks, trench motion, and net lithospheric rotation. An interesting outcome of our analysis is that the geodynamic reference frame proposed by Tetley et al. (2019) — designed to improve absolute plate motion models by integrating multiple constraints rather than relying on a single observation, such as hotspot tracks in the S2012 frame — supports our conclusion that lower mantle anomalies are not solely composed of subducted slabs.

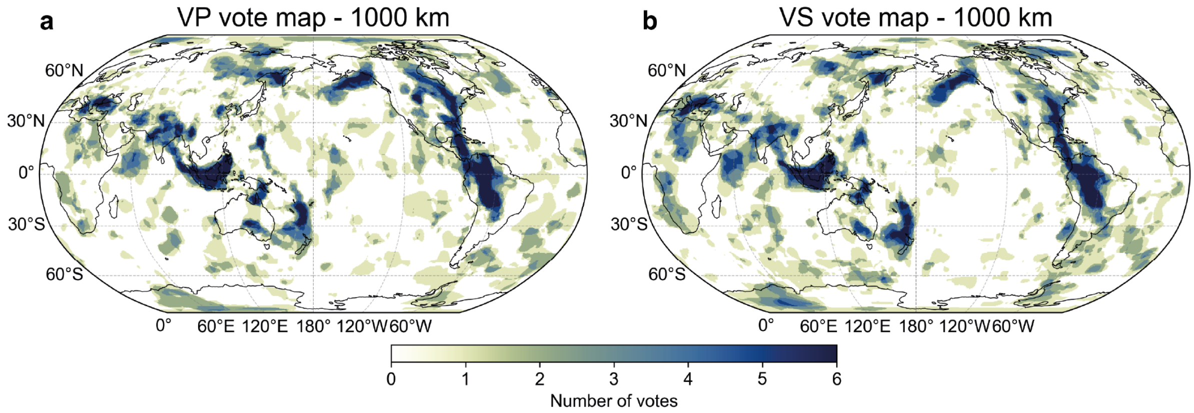

Figure S2: **Vote maps of positive wave speed anomalies at 1000 km depth in travel-time tomographic models.** Vote maps constructed using SubMachine (Hosseini et al., 2018) with default vote map parameters such that positive anomalies are defined as $>0.0\%\ dV$. (a) Vote map of six P-wave speed models. References given in Table S2. (b) Vote map of six P-wave speed models. References given in Table S3

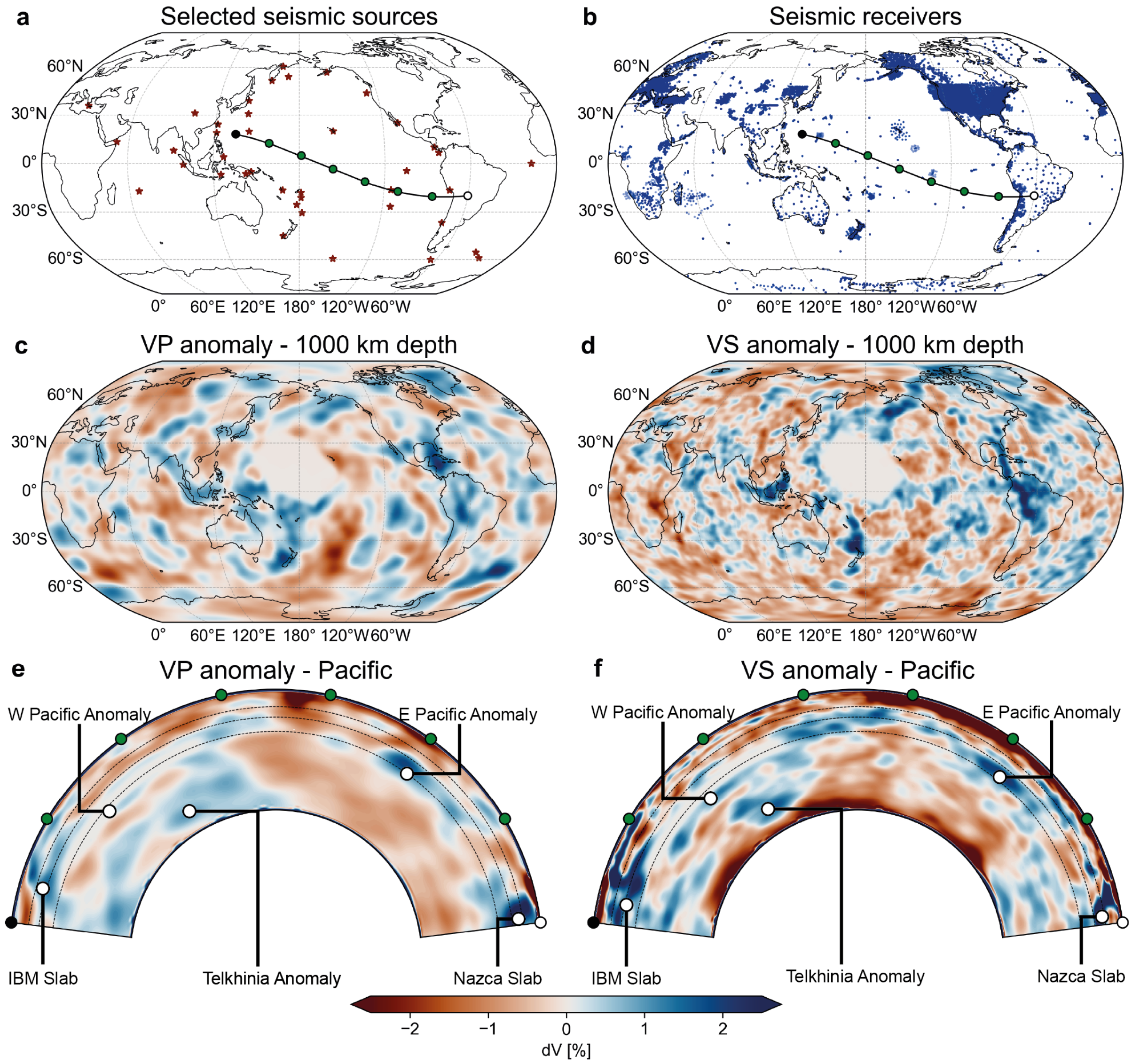

Figure S3: **Simulated sources and copy of REVEAL with western Pacific anomaly removed.** Panels (a-b) show the sources and receivers and the trace of the cross section in panels (e-f). Panels (c-d) show the VP an VS anomalies at 1000 km depth, and panels (e-f) show the same anomalies for a cross section through the Pacific Ocean. These panels are to be compared to panels (c-f) of Fig. 1 in the main text (where the western Pacific anomaly is included).

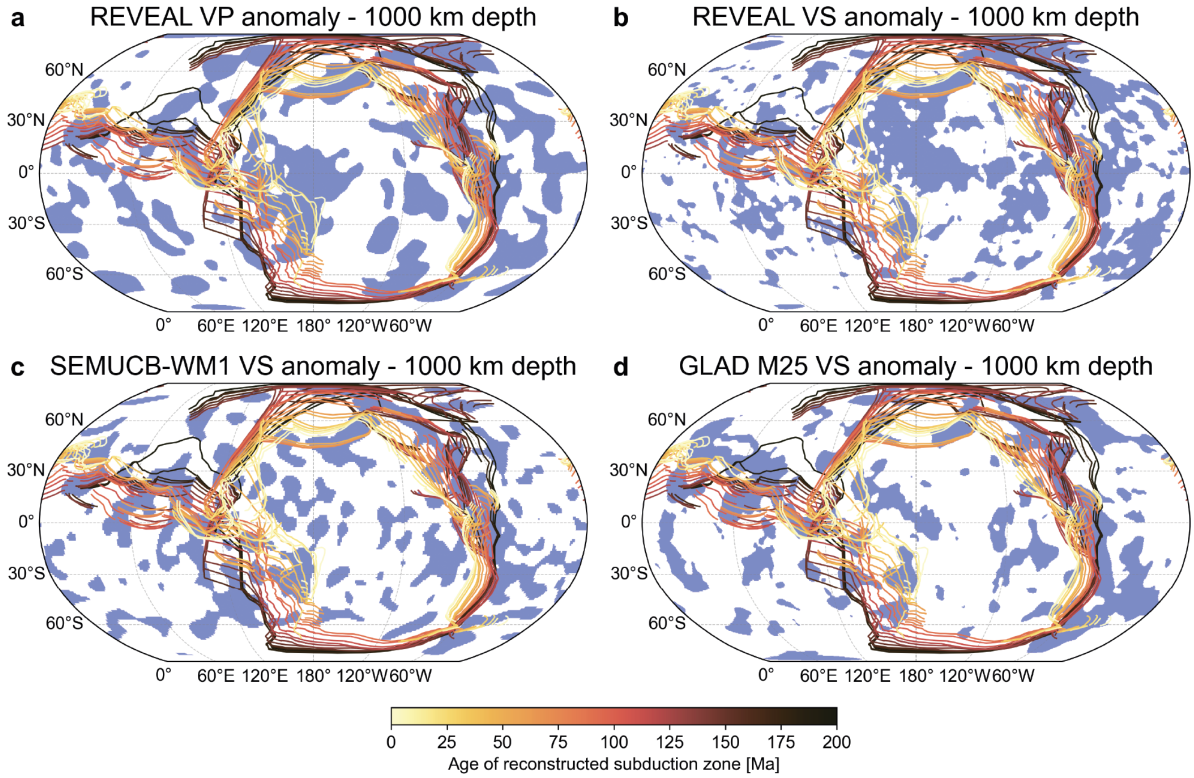

Figure S4: **Reconstructed subduction zones of the C2020 reconstruction over positive anomalies in global FWI models.** This figure illustrates the spatial distribution of reconstructed subduction zones for the C2020 in the last 200 Ma compared to the distribution of positive wave speed anomalies at 1000 km depth (in light blue, defined as $\geq 0.1\ dVP$ and $\geq 0.2\ dVS$ (Clennett et al., 2020; Wu et al., 2016; van der Meer et al., 2018)) in three global FWI models (Thrastarson et al., 2024; Lei et al., 2020; French and Romanowicz, 2014).

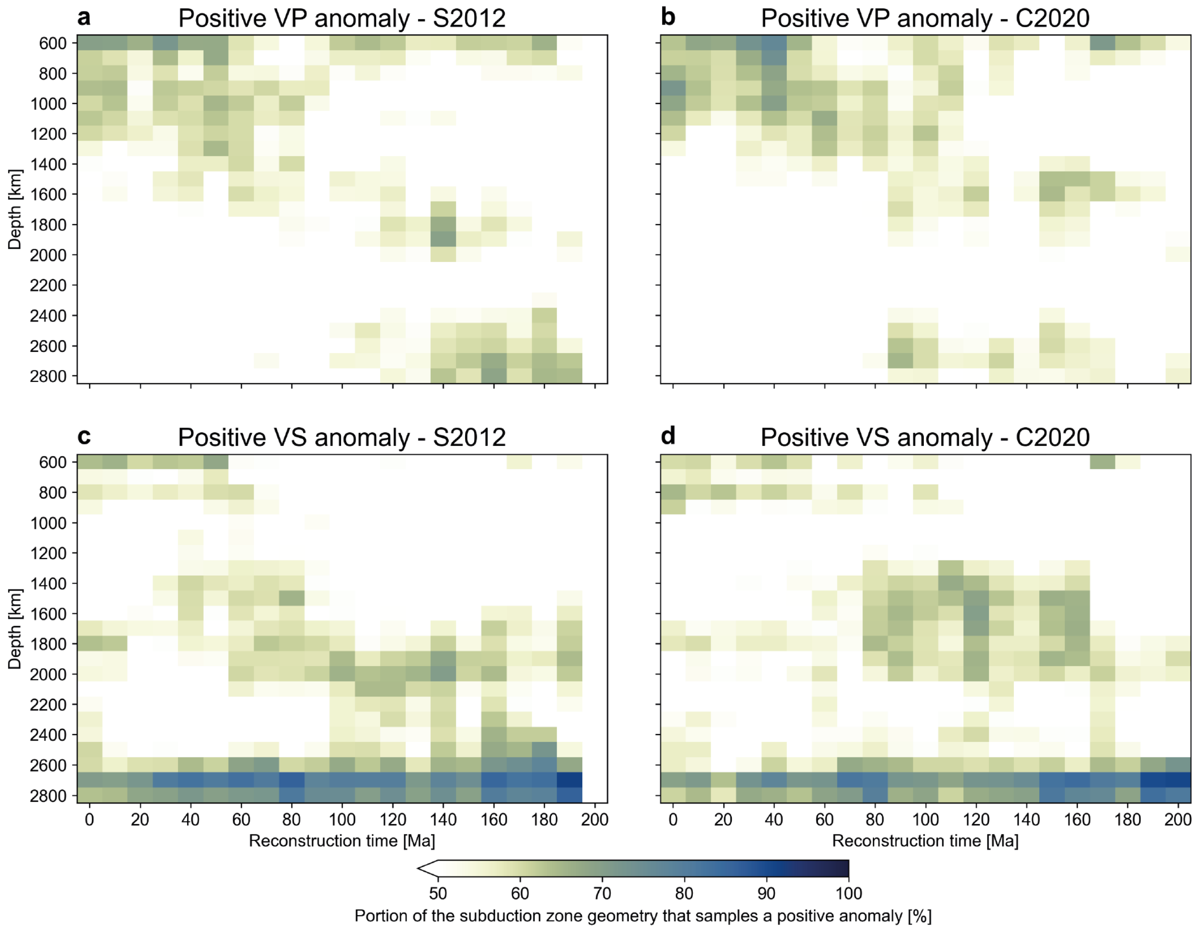

Figure S5: **Correlation between subduction zones and seismic wave speed anomalies with a 200 km sampling distance.** This figure shows the same information as Fig. 6 in the main text, but with anomalies sampled 200 km (the typical arc-trench distance (van Hinsbergen and Schouten, 2021)) inboard of reconstructed subduction zones.

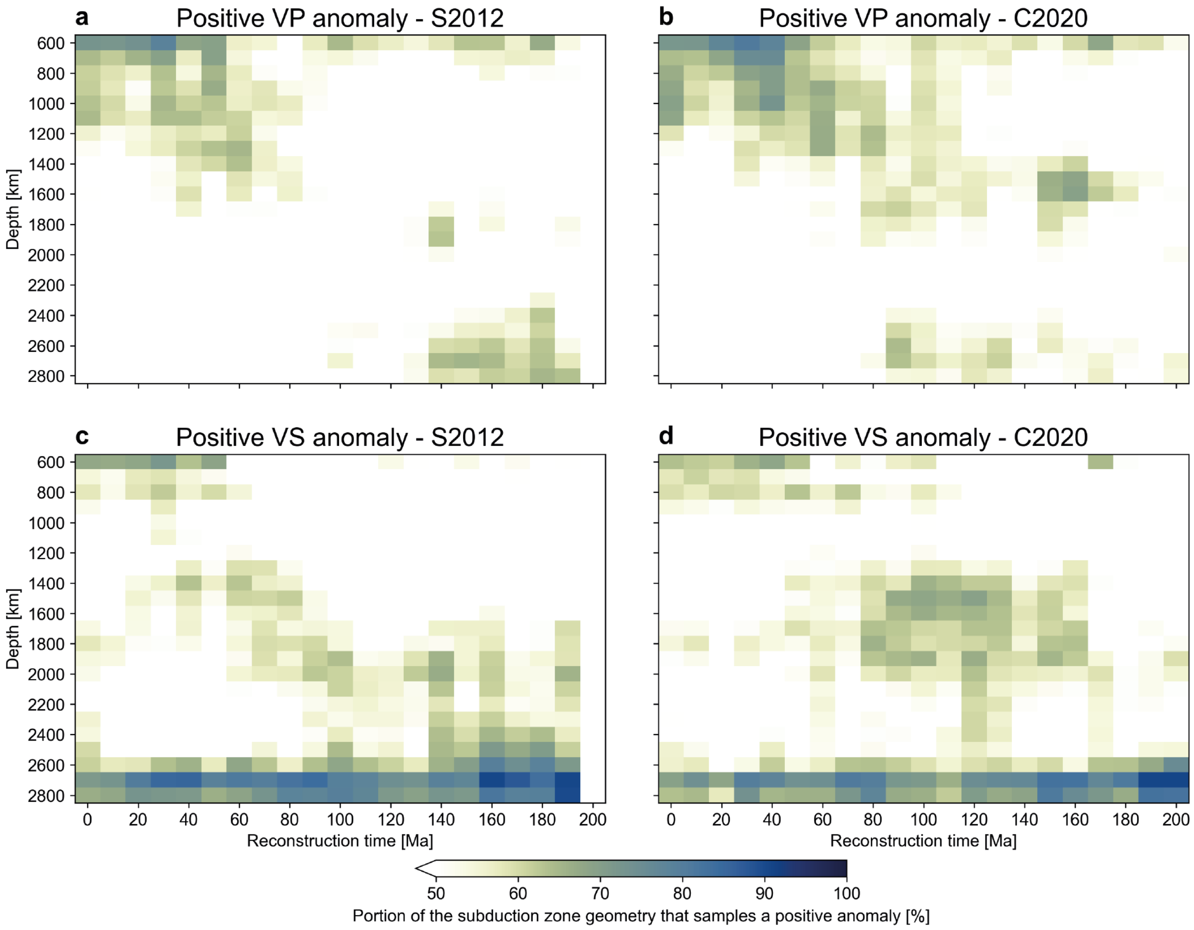

Figure S6: **Correlation between subduction zones and seismic wave speed anomalies with a 400 km sampling distance.** This figure shows the same information as Fig. 6 in the main text, but with anomalies sampled 400 km (the typical arc-trench distance (van Hinsbergen and Schouten, 2021)) inboard of reconstructed subduction zones.

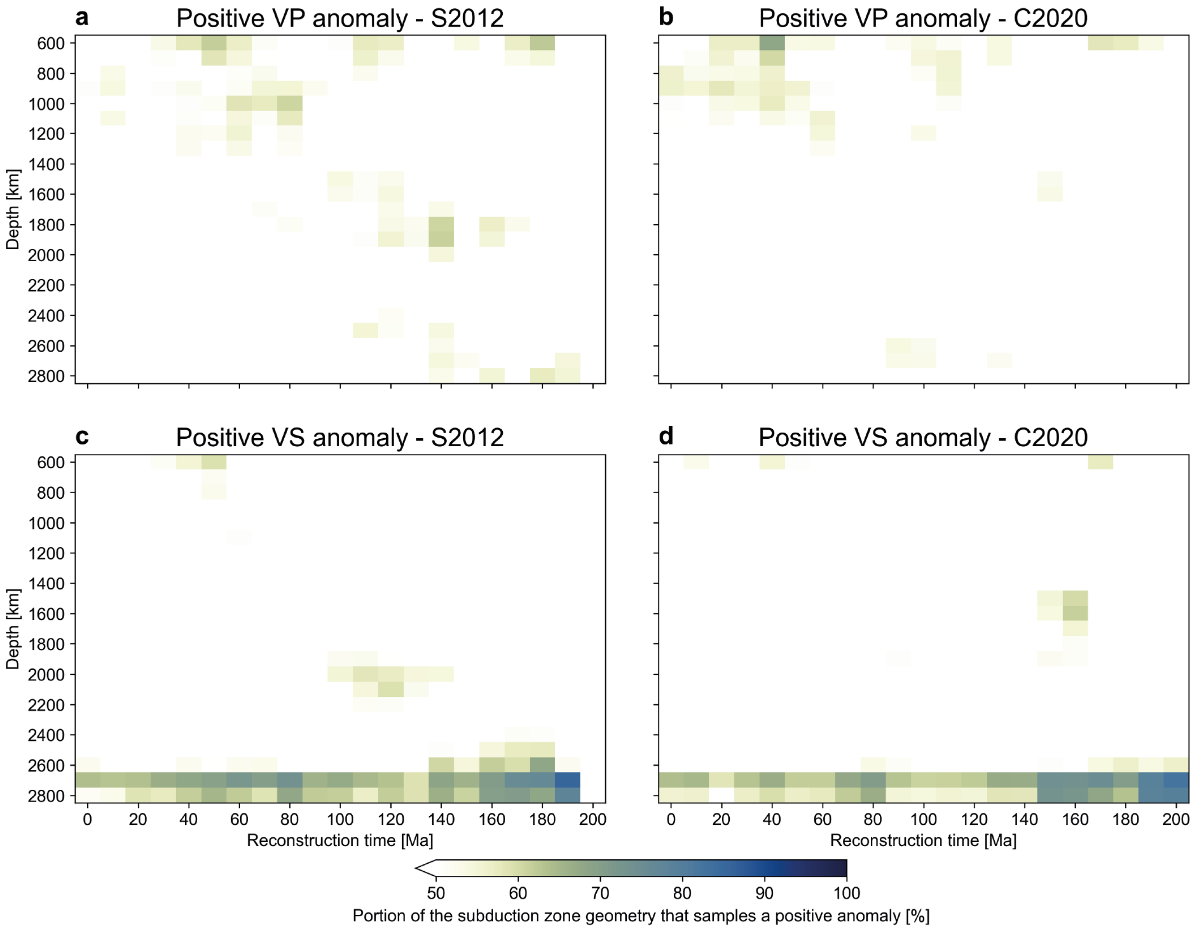

Figure S7: **Correlation between subduction zones and seismic wave speed anomalies with a doubled velocity anomaly threshold.** This figure shows the same information as Fig. 6 in the main text, but with positive anomalies defined as $\geq 0.2\ dVP$ and $\geq 0.4\ dVS$.

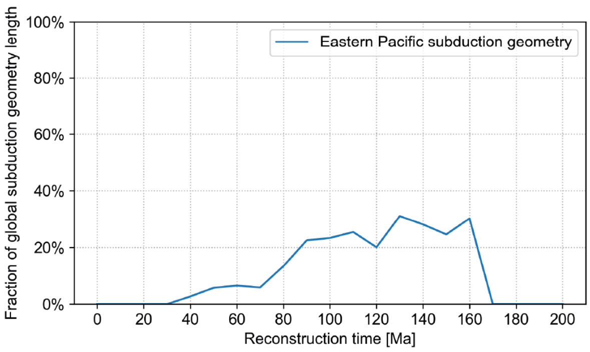

Figure S8: Length of the subduction zones in the East Pacific Basin in the C2020 reconstruction as a fraction of the total global subduction geometry length.

References

Advokaat, E. L. and van Hinsbergen, D. J. J. (2023). Finding Argoland: Reconstructing a microcontinental archipelago from the SE Asian accretionary orogen. *Gondwana Research*.

Amaru, M. L. (2007). Global travel time tomography with 3-D reference models. *Geologica Ultraiectina*, 274. Accepted: 2007-02-02T14:51:52Z ISBN: 9789057441394 Publisher: Utrecht University.

Auer, L., Boschi, L., Becker, T. W., Nissen-Meyer, T., and Giardini, D. (2014). Savani: A variable resolution whole-mantle model of anisotropic shear velocity variations based on multiple data sets. *Journal of Geophysical Research: Solid Earth*, 119(4):3006–3034. _eprint: https://onlinelibrary.wiley.com/doi/pdf/10.1002/2013JB010773.

Barber, A. J. and Crow, M. J. (2009). Structure of Sumatra and its implications for the tectonic assembly of Southeast Asia and the destruction of Paleotethys. *Island Arc*, 18(1):3–20. _eprint: https://onlinelibrary.wiley.com/doi/pdf/10.1111/j.1440-1738.2008.00631.x.

Bazhenov, M. L., Zharov, A. E., Levashova, N. M., Kodama, K., Bragin, N. Y., Fedorov, P. I., Bragina, L. G., and Lyapunov, S. M. (2001). Paleomagnetism of a Late Cretaceous island arc complex from South Sakhalin, East Asia: Convergent boundaries far away from the Asian continental margin? *Journal of Geophysical Research: Solid Earth*, 106(B9):19193–19205. _eprint: https://onlinelibrary.wiley.com/doi/pdf/10.1029/2000JB900458.

Beck, R. A., Burbank, D. W., Sercombe, W. J., Khan, A. M., and Lawrence, R. D. (1996). Late Cretaceous ophiolite obduction and Paleocene India-Asia collision in the westernmost Himalaya. *Geodinamica Acta*, 9(2-3):114–144. Place: Abingdon Publisher: Taylor & Francis Ltd WOS:A1996UT44800004.

Becker, T. W. (2006). On the effect of temperature and strain-rate dependent viscosity on global mantle flow, net rotation, and plate-driving forces. *Geophysical Journal International*, 167(2):943–957.

Becker, T. W. and Boschi, L. (2002). A comparison of tomographic and geodynamic mantle models. *Geochemistry, Geophysics, Geosystems*, 3(1). _eprint: https://onlinelibrary.wiley.com/doi/pdf/10.1029/2001GC000168.

Becker, T. W. and Faccenna, C. (2011). Mantle conveyor beneath the Tethyan collisional belt. *Earth and Planetary Science Letters*, 310(3):453–461.

Becker, T. W. and O'Connell, R. J. (2001). Predicting plate velocities with mantle circulation models. *Geochemistry, Geophysics, Geosystems*, 2(12). _eprint: https://onlinelibrary.wiley.com/doi/pdf/10.1029/2001GC000171.

Boschman, L. M., Molina Garza, R. S., Langereis, C. G., and van Hinsbergen, D. J. (2018). Paleomagnetic constraints on the kinematic relationship between the Guerrero terrane (Mexico) and North America since Early Cretaceous time. *GSA Bulletin*, 130(7-8):1131–1142.

Boschman, L. M., van Hinsbergen, D. J. J., Torsvik, T. H., Spakman, W., and Pindell, J. L. (2014). Kinematic reconstruction of the Caribbean region since the Early Jurassic. *Earth-Science Reviews*, 138:102–136.

Burdick, S., Vernon, F. L., Martynov, V., Eakins, J., Cox, T., Tytell, J., Mulder, T., White, M. C., Astiz, L., Pavlis, G. L., and van der Hilst, R. D. (2017). Model Update May 2016: Upper-Mantle Heterogeneity beneath North America from Travel-Time Tomography with Global and USArray Data. *Seismological Research Letters*, 88(2A):319–325.

Chen, Y.-W., Wu, J., and Suppe, J. (2019). Southward propagation of Nazca subduction along the Andes. *Nature*, 565(7740):441–447. Number: 7740 Publisher: Nature Publishing Group.

Clennett, E. J., Sigloch, K., Mihalynuk, M. G., Seton, M., Henderson, M. A., Hosseini, K., Mohammadzaheri, A., Johnston, S. T., and Müller, R. D. (2020). A Quantitative Tomotectonic Plate Reconstruction of Western North America and the Eastern Pacific Basin. *Geochemistry, Geophysics, Geosystems*, 21(8):e2020GC009117. _eprint: https://onlinelibrary.wiley.com/doi/pdf/10.1029/2020GC009117.

Conrad, C. P. and Behn, M. D. (2010). Constraints on lithosphere net rotation and asthenospheric viscosity from global mantle flow models and seismic anisotropy. *Geochemistry, Geophysics, Geosystems*, 11(5). _eprint: https://onlinelibrary.wiley.com/doi/pdf/10.1029/2009GC002970.

Conrad, C. P., Lithgow-Bertelloni, C., and Louden, K. E. (2004). Iceland, the Farallon slab, and dynamic topography of the North Atlantic. *Geology*, 32(3):177–180.

Crotwell, H. P., Owens, T. J., and Ritsema, J. (1999). The TauP Toolkit: Flexible Seismic Travel-time and Ray-path Utilities. *Seismological Research Letters*, 70(2):154–160.

Davies, D. R., Goes, S., and Lau, H. C. P. (2015). Thermally Dominated Deep Mantle LLSVPs: A Review. In Khan, A. and Deschamps, F., editors, *The Earth's Heterogeneous Mantle: A Geophysical, Geodynamical, and Geochemical Perspective*, pages 441–477. Springer International Publishing, Cham.

Deschamps, F., Li, Y., and Tackley, P. J. (2015). Large-Scale Thermo-chemical Structure of the Deep Mantle: Observations and Models. In Khan, A. and Deschamps, F., editors, *The Earth's Heterogeneous Mantle: A Geophysical, Geodynamical, and Geochemical Perspective*, Springer Geophysics, pages 479–515. Springer International Publishing, Cham.

Deschamps, F. and Trampert, J. (2003). Mantle tomography and its relation to temperature and composition. *Physics of the Earth and Planetary Interiors*, 140(4):277–291.

Domeier, M., Doubrovine, P. V., Torsvik, T. H., Spakman, W., and Bull, A. L. (2016). Global correlation of lower mantle structure and past subduction. *Geophysical Research Letters*, 43(10):4945–4953. _eprint: https://onlinelibrary.wiley.com/doi/pdf/10.1002/2016GL068827.

Domeier, M., Shephard, G. E., Jakob, J., Gaina, C., Doubrovine, P. V., and Torsvik, T. H. (2017). Intraoceanic subduction spanned the Pacific in the Late Cretaceous–Paleocene. *Science Advances*, 3(11):eaao2303. Publisher: American Association for the Advancement of Science.

Doubrovine, P. V., Steinberger, B., and Torsvik, T. H. (2012). Absolute plate motions in a reference frame defined by moving hot spots in the Pacific, Atlantic, and Indian oceans. *Journal of Geophysical Research: Solid Earth*.

Dziewonski, A. M. and Anderson, D. L. (1981). Preliminary reference Earth model. *Physics of the Earth and Planetary Interiors*, 25(4):297–356.

Eagles, G. and Jokat, W. (2014). Tectonic reconstructions for paleobathymetry in Drake Passage. *Tectonophysics*, 611:28–50.

Engebretson, D. C., Cox, A., and Gordon, R. G. (1985). Relative Motions Between Oceanic and Continental Plates in the Pacific Basin. *GSA Special Papers*, 206.

Faccenna, C. and Becker, T. W. (2010). Shaping mobile belts by small-scale convection. *Nature*, 465(7298):602–605. Publisher: Nature Publishing Group.

Fichtner, A. and Trampert, J. (2011). Resolution analysis in full waveform inversion. *Geophysical Journal International*, 187(3):1604–1624.

French, S. W. and Romanowicz, B. A. (2014). Whole-mantle radially anisotropic shear velocity structure from spectral-element waveform tomography. *Geophysical Journal International*, 199(3):1303–1327.

Fuston, S. and Wu, J. (2020). Raising the Resurrection plate from an unfolded-slab plate tectonic reconstruction of northwestern North America since early Cenozoic time. *GSA Bulletin*, 133(5-6):1128–1140.

Gaina, C., Van Hinsbergen, D. J., and Spakman, W. (2015). Tectonic interactions between India and Arabia since the Jurassic reconstructed from marine geophysics, ophiolite geology, and seismic tomography. *Tectonics*, 34(5):875–906. Publisher: John Wiley & Sons, Ltd.

Gerya, T. (2019). *Introduction to Numerical Geodynamic Modelling*. Cambridge University Press. Google-Books-ID: 8XGSDwAAQBAJ.

Ghosh, A., Becker, T. W., and Zhong, S. J. (2010). Effects of lateral viscosity variations on the geoid. *Geophysical Research Letters*, 37(1). _eprint: https://onlinelibrary.wiley.com/doi/pdf/10.1029/2009GL040426.

Gibbons, A. D., Zahirovic, S., Müller, R. D., Whittaker, J. M., and Yatheesh, V. (2015). A tectonic model reconciling evidence for the collisions between India, Eurasia and intra-oceanic arcs of the central-eastern Tethys. *Gondwana Research*, 28(2):451–492.

Grand, S. P. (2002). Mantle shear–wave tomography and the fate of subducted slabs. *Philosophical Transactions of the Royal Society of London. Series A: Mathematical, Physical and Engineering Sciences*, 360(1800):2475–2491. Publisher: Royal Society.

Hall, R. (2012). Late Jurassic–Cenozoic reconstructions of the Indonesian region and the Indian Ocean. *Tectonophysics*, 570-571:1–41.

Heine, C., Zoethout, J., and Müller, R. D. (2013). Kinematics of the South Atlantic rift. *Solid Earth*, 4(2):215–253. Publisher: Copernicus GmbH.

Hosseini, K., Matthews, K. J., Sigloch, K., Shephard, G. E., Domeier, M., and Tsekhmistrenko, M. (2018). SubMachine: Web-Based Tools for Exploring Seismic Tomography and Other Models of Earth's Deep Interior - Hosseini - 2018 - - Wiley Online Library. *Geochemistry, Geophysics, Geosystems*.

Hosseini, K., Sigloch, K., Tsekhmistrenko, M., Zaheri, A., Nissen-Meyer, T., and Igel, H. (2020). Global mantle structure from multifrequency tomography using P, PP and P-diffracted waves. *Geophysical Journal International*, 220(1):96–141.

Houser, C., Masters, G., Shearer, P., and Laske, G. (2008). Shear and compressional velocity models of the mantle from cluster analysis of long-period waveforms. *Geophysical Journal International*, 174(1):195–212.

Johnson, S. E., Tate, M. C., and Mark Fanning, C. (1999). New geologic mapping and SHRIMP U-Pb zircon data in the Peninsular Ranges batholith, Baja California, Mexico: Evidence for a suture? *Geology*, 27(8):743–746.

Johnston, S. T. (2001). The Great Alaskan Terrane Wreck: reconciliation of paleomagnetic and geological data in the northern Cordillera. *Earth and Planetary Science Letters*, 193(3):259–272.

Kennett, B. L. N. and Engdahl, E. R. (1991). Traveltimes for global earthquake location and phase identification. *Geophysical Journal International*, 105(2):429–465.

Khan, M. A., Kerr, A. C., and Mahmood, K. (2007). Formation and tectonic evolution of the Cretaceous–Jurassic Muslim Bagh ophiolitic complex, Pakistan: Implications for the composite tectonic setting of ophiolites. *Journal of Asian Earth Sciences*, 32:112–127.

Konstantinovskaia, E. A. (2001). Arc–continent collision and subduction reversal in the Cenozoic evolution of the Northwest Pacific: an example from Kamchatka (NE Russia). *Tectonophysics*, 333(1):75–94.

Krischer, L., Megies, T., Barsch, R., Beyreuther, M., Lecocq, T., Caudron, C., and Wassermann, J. (2015). ObsPy: a bridge for seismology into the scientific Python ecosystem. *Computational Science & Discovery*, 8(1):014003. Publisher: IOP Publishing.

Larson, R. L. and Chase, C. G. (1972). Late Mesozoic Evolution of the Western Pacific Ocean. *GSA Bulletin*, 83(12):3627–3644.

Lei, W., Ruan, Y., Bozdağ, E., Peter, D., Lefebvre, M., Komatitsch, D., Tromp, J., Hill, J., Podhorszki, N., and Pugmire, D. (2020). Global adjoint tomography—model GLAD-M25. *Geophysical Journal International*, 223(1):1–21.

Li, C., van der Hilst, R. D., Engdahl, E. R., and Burdick, S. (2008). A new global model for P wave speed variations in Earth's mantle. *Geochemistry, Geophysics, Geosystems*, 9(5). _eprint: https://onlinelibrary.wiley.com/doi/pdf/10.1029/2007GC001806.

Lin, Y.-A., Colli, L., and Wu, J. (2022). NW Pacific-Panthalassa Intra-Oceanic Subduction During Mesozoic Times From Mantle Convection and Geoid Models. *Geochemistry, Geophysics, Geosystems*, 23(11):e2022GC010514. _eprint: https://onlinelibrary.wiley.com/doi/pdf/10.1029/2022GC010514.

Lévêque, J.-J., Rivera, L., and Wittlinger, G. (1993). On the use of the checker-board test to assess the resolution of tomographic inversions. *Geophysical Journal International*, 115(1):313–318.

Martin, C. R., Jagoutz, O., Upadhyay, R., Royden, L. H., Eddy, M. P., Bailey, E., Nichols, C. I. O., and Weiss, B. P. (2020). Paleocene latitude of the Kohistan–Ladakh arc indicates multistage India–Eurasia collision. *Proceedings of the National Academy of Sciences*, 117(47):29487–29494. Publisher: Proceedings of the National Academy of Sciences.

Matthews, K. J., Maloney, K. T., Zahirovic, S., Williams, S. E., Seton, M., and Müller, R. D. (2016). Global plate boundary evolution and kinematics since the late Paleozoic. *Global and Planetary Change*, 146:226–250.

McKenzie, D. P. and Parker, R. L. (1967). The North Pacific: an Example of Tectonics on a Sphere. *Nature*, 216(5122):1276–1280. Number: 5122 Publisher: Nature Publishing Group.

McNamara, A. K. and Zhong, S. (2005). Thermochemical structures beneath Africa and the Pacific Ocean. *Nature*, 437(7062):1136–1139. Number: 7062 Publisher: Nature Publishing Group.

Mohammadzaheri, A., Sigloch, K., Hosseini, K., and Mihalynuk, M. G. (2021). Subducted Lithosphere Under South America From Multifrequency P Wave Tomography. *Journal of Geophysical Research: Solid Earth*, 126(6):e2020JB020704. _eprint: https://onlinelibrary.wiley.com/doi/pdf/10.1029/2020JB020704.

Montelli, R., Nolet, G., Dahlen, F. A., and Masters, G. (2006). A catalogue of deep mantle plumes: New results from finite-frequency tomography. *Geochemistry, Geophysics, Geosystems*, 7(11). _eprint: https://onlinelibrary.wiley.com/doi/pdf/10.1029/2006GC001248.

Montelli, R., Nolet, G., Masters, G., Dahlen, F. A., and Hung, S.-H. (2004). Global P and PP traveltime tomography: rays versus waves. *Geophysical Journal International*, 158(2):637–654.

Müller, R. D., Roest, W. R., Royer, J. Y., Gahagan, L. M., and Sclater, J. G. (1997). Digital isochrons of the world's ocean floor. *Journal of Geophysical Research: Solid Earth*, 102(B2):3211–3214. Publisher: John Wiley & Sons, Ltd.

Müller, R. D., Sdrolias, M., Gaina, C., Steinberger, B., and Heine, C. (2008). Long-Term Sea-Level Fluctuations Driven by Ocean Basin Dynamics. *Science*, 319(5868):1357–1362. Publisher: American Association for the Advancement of Science.

Müller, R. D., Zahirovic, S., Williams, S. E., Cannon, J., Seton, M., Bower, D. J., Tetley, M. G., Heine, C., Le Breton, E., Liu, S., Russell, S. H. J., Yang, T., Leonard, J., and Gurnis, M. (2019). A Global Plate Model Including Lithospheric Deformation Along Major Rifts and Orogens Since the Triassic. *Tectonics*, 38(6):1884–1907. _eprint: https://onlinelibrary.wiley.com/doi/pdf/10.1029/2018TC005462.

Nerlich, R., Clark, S. R., and Bunge, H.-P. (2013). The Scotia Sea gateway: No outlet for Pacific mantle. *Tectonophysics*, 604:41–50.

Nokleberg, W. J., Scholl, D. W., Parfenov, L. M., Monger, J. W. H., Norton, I. O., Khanchuk, A. I., Stone, D. B., Scotese, C. R., and Fujita, K. (2001). Phanerozoic tectonic evolution of the Circum-North Pacific. *U.S. Geological Survey*, Professional Paper 1626.

Obayashi, M., Yoshimitsu, J., Nolet, G., Fukao, Y., Shiobara, H., Sugioka, H., Miyamachi, H., and Gao, Y. (2013). Finite frequency whole mantle P wave tomography: Improvement of subducted slab images. *Geophysical Research Letters*, 40(21):5652–5657. _eprint: https://onlinelibrary.wiley.com/doi/pdf/10.1002/2013GL057401.

O'Neill, C., Müller, D., and Steinberger, B. (2005). On the uncertainties in hot spot reconstructions and the significance of moving hot spot reference frames. *Geochemistry, Geophysics, Geosystems*, 6(4). _eprint: https://onlinelibrary.wiley.com/doi/pdf/10.1029/2004GC000784.

Rahimzadeh Bajgiran, M., Colli, L., and Wu, J. (2023). Assessing large-scale mantle compositional heterogeneity from machine learning analysis of 28 global P- and S-wave tomography models. *Geophysical Journal International*, 235(3):2778–2793.

Ritsema, J., Deuss, A., van Heijst, H. J., and Woodhouse, J. H. (2011). S40RTS: a degree-40 shear-velocity model for the mantle from new Rayleigh wave dispersion, teleseismic traveltime and normal-mode splitting function measurements. *Geophysical Journal International*, 184(3):1223–1236.

Schuberth, B. S. A., Bunge, H.-P., and Ritsema, J. (2009). Tomographic filtering of high-resolution mantle circulation models: Can seismic heterogeneity be explained by temperature alone? *Geochemistry, Geophysics, Geosystems*, 10(5). _eprint: https://onlinelibrary.wiley.com/doi/pdf/10.1029/2009GC002401.

Seton, M., Müller, R. D., Zahirovic, S., Gaina, C., Torsvik, T., Shephard, G., Talsma, A., Gurnis, M., Turner, M., Maus, S., and Chandler, M. (2012). Global continental and ocean basin reconstructions since 200Ma. *Earth-Science Reviews*, 113(3):212–270.

Seton, M., Williams, S. E., Domeier, M., Collins, A. S., and Sigloch, K. (2023). Deconstructing plate tectonic reconstructions. *Nature Reviews Earth & Environment*, pages 1–20. Publisher: Nature Publishing Group.

Shephard, G. E., Matthews, K. J., Hosseini, K., and Domeier, M. (2017). On the consistency of seismically imaged lower mantle slabs | Scientific Reports. *Nature*.

Shephard, G. E., Müller, R. D., and Seton, M. (2013). The tectonic evolution of the Arctic since Pangea breakup: Integrating constraints from surface geology and geophysics with mantle structure. *Earth-Science Reviews*, 124:148–183.

Sigloch, K. and Mihalynuk, M. G. (2013). Intra-oceanic subduction shaped the assembly of Cordilleran North America. *Nature*, 496(7443):50–56. Number: 7443 Publisher: Nature Publishing Group.

Sigloch, K. and Mihalynuk, M. G. (2017). Mantle and geological evidence for a Late Jurassic–Cretaceous suture spanning North America. *GSA Bulletin*, 129(11-12):1489–1520.

Simmons, N. A., Forte, A. M., Boschi, L., and Grand, S. P. (2010). GyPSuM: A joint tomographic model of mantle density and seismic wave speeds. *Journal of Geophysical Research: Solid Earth*, 115(B12). _eprint: https://onlinelibrary.wiley.com/doi/pdf/10.1029/2010JB007631.

Simmons, N. A., Myers, S. C., Johannesson, G., and Matzel, E. (2012). LLNL-G3Dv3: Global P wave tomography model for improved regional and teleseismic travel time prediction. *Journal of Geophysical Research: Solid Earth*, 117(B10). _eprint: https://onlinelibrary.wiley.com/doi/pdf/10.1029/2012JB009525.

Stampfli, G. M. and Borel, G. D. (2002). A plate tectonic model for the Paleozoic and Mesozoic constrained by dynamic plate boundaries and restored synthetic oceanic isochrons. *Earth and Planetary Science Letters*, 196(1):17–33.

Steinberger, B. and Calderwood, A. R. (2006). Models of large-scale viscous flow in the Earth's mantle with constraints from mineral physics and surface observations. *Geophysical Journal International*, 167(3):1461–1481.

Steinberger, B. and Torsvik, T. H. (2012). A geodynamic model of plumes from the margins of Large Low Shear Velocity Provinces. *Geochemistry, Geophysics, Geosystems*, 13(1). _eprint: https://onlinelibrary.wiley.com/doi/pdf/10.1029/2011GC003808.

Straume, E. O., Steinberger, B., Becker, T. W., and Faccenna, C. (2024). Impact of mantle convection and dynamic topography on the Cenozoic paleogeography of Central Eurasia and the West Siberian Seaway. *Earth and Planetary Science Letters*, 630:118615.

Tetley, M. G., Williams, S. E., Gurnis, M., Flament, N., and Müller, R. D. (2019). Constraining Absolute Plate Motions Since the Triassic. *Journal of Geophysical Research: Solid Earth*, 124(7):7231–7258. _eprint: https://onlinelibrary.wiley.com/doi/pdf/10.1029/2019JB017442.

Thrastarson, S., van Herwaarden, D., Noe, S., Josef Schiller, C., and Fichtner, A. (2024). REVEAL: A Global Full-Waveform Inversion Model. *Bulletin of the Seismological Society of America*, 114(3):1392–1406.

Torsvik, T. H., Steinberger, B., Gurnis, M., and Gaina, C. (2010). Plate tectonics and net lithosphere rotation over the past 150My. *Earth and Planetary Science Letters*, 291(1):106–112.

Torsvik, T. H., Steinberger, B., Shephard, G. E., Doubrovine, P. V., Gaina, C., Domeier, M., Conrad, C. P., and Sager, W. W. (2019). Pacific-Panthalassic Reconstructions: Overview, Errata and the Way Forward. *Geochemistry, Geophysics, Geosystems*, 20(7):3659–3689. _eprint: https://onlinelibrary.wiley.com/doi/pdf/10.1029/2019GC008402.

Trampert, J. and Spetzler, J. (2006). Surface wave tomography: finite-frequency effects lost in the null space. *Geophysical Journal International*, 164(2):394–400.

Trampert, J., Vacher, P., and Vlaar, N. (2001). Sensitivities of seismic velocities to temperature, pressure and composition in the lower mantle. *Physics of the Earth and Planetary Interiors*, 124(3):255–267.

Vaes, B., van Hinsbergen, D. J. J., and Boschman, L. M. (2019). Reconstruction of Subduction and Back-Arc Spreading in the NW Pacific and Aleutian Basin: Clues to Causes of Cretaceous and Eocene Plate Reorganizations. *Tectonics*, 38(4):1367–1413. _eprint: https://onlinelibrary.wiley.com/doi/pdf/10.1029/2018TC005164.

van de Lagemaat, S. H. A., Swart, M. L. A., Vaes, B., Kosters, M. E., Boschman, L. M., Burton-Johnson, A., Bijl, P. K., Spakman, W., and van Hinsbergen, D. J. J. (2021). Subduction initiation in the Scotia Sea region and opening of the Drake Passage: When and why? *Earth-Science Reviews*, 215:103551.

van de Lagemaat, S. H. A. and van Hinsbergen, D. J. J. (2023). Plate tectonic cross-roads: Reconstructing the Panthalassa-Neotethys Junction Region from Philippine Sea Plate and Australasian oceans and orogens. *Gondwana Research*.

van der Meer, D. G., Spakman, W., van Hinsbergen, D. J. J., Amaru, M. L., and Torsvik, T. H. (2010). Towards absolute plate motions constrained by lower-mantle slab remnants. *Nature Geoscience*, 3(1):36–40. Number: 1 Publisher: Nature Publishing Group.

van der Meer, D. G., Torsvik, T. H., Spakman, W., van Hinsbergen, D. J. J., and Amaru, M. L. (2012). Intra-Panthalassa Ocean subduction zones revealed by fossil arcs and mantle structure. *Nature Geoscience*, 5(3):215–219. Number: 3 Publisher: Nature Publishing Group.

van der Meer, D. G., van Hinsbergen, D. J. J., and Spakman, W. (2018). Atlas of the underworld: Slab remnants in the mantle, their sinking history, and a new outlook on lower mantle viscosity. *Tectonophysics*, 723:309–448.

van Herwaarden, D.-P., Thrastarson, S., Hapla, V., Afanasiev, M., Trampert, J., and Fichtner, A. (2023). Full-Waveform Tomography of the African Plate Using Dynamic Mini-Batches. *Journal of Geophysical Research: Solid Earth*, 128(6):e2022JB026023. _eprint: https://onlinelibrary.wiley.com/doi/pdf/10.1029/2022JB026023.

van Hinsbergen, D. J. J. and Schouten, T. L. A. (2021). Deciphering paleogeography from orogenic architecture: Constructing orogens in a future supercontinent as thought experiment. *American Journal of Science*, 321(6):955–1031. Publisher: American Journal of Science Section: Article.

van Hinsbergen, D. J. J., Torsvik, T. H., Schmid, S. M., Maţenco, L. C., Maffione, M., Vissers, R. L. M., Gürer, D., and Spakman, W. (2020). Orogenic architecture of the Mediterranean region and kinematic reconstruction of its tectonic evolution since the Triassic. *Gondwana Research*, 81:79–229.

Westerweel, J., Roperch, P., Licht, A., Dupont-Nivet, G., Win, Z., Poblete, F., Ruffet, G., Swe, H. H., Thi, M. K., and Aung, D. W. (2019). Burma Terrane part of the Trans-Tethyan arc during collision with India according to palaeomagnetic data. *Nature Geoscience*, 12(10):863–868. Number: 10 Publisher: Nature Publishing Group.

Wetmore, P. H., Hughes, S. S., Stremtan, C., Ducea, M. N., and Alsleben, H. (2014). Tectonic implications of postcontractional magmatism of the Alisitos arc segment of the Peninsular Ranges, Baja California, Mexico. In *Peninsular Ranges Batholith, Baja California and Southern California*. Geological Society of America.

Wu, J., Lin, Y.-A., Flament, N., Wu, J. T.-J., and Liu, Y. (2022). Northwest Pacific-Izanagi plate tectonics since Cretaceous times from western Pacific mantle structure. *Earth and Planetary Science Letters*, 583:117445.

Wu, J. and Suppe, J. (2018). Proto-South China Sea Plate Tectonics Using Subducted Slab Constraints from Tomography. *Journal of Earth Science*, 29(6):1304–1318.

Wu, J., Suppe, J., Lu, R., and Kanda, R. (2016). Philippine Sea and East Asian plate tectonics since 52 Ma constrained by new subducted slab reconstruction methods. *Journal of Geophysical Research: Solid Earth*, 121(6):4670–4741. _eprint: https://onlinelibrary.wiley.com/doi/pdf/10.1002/2016JB012923.

Yomogida, K. (1992). Fresnel zone inversion for lateral heterogeneities in the earth. *pure and applied geophysics*, 138(3):391–406.

Zahirovic, S., Seton, M., and Müller, R. D. (2014). The Cretaceous and Cenozoic tectonic evolution of Southeast Asia. *Solid Earth*, 5(1):227–273. Publisher: Copernicus GmbH.

Zhong, S. (2001). Role of ocean-continent contrast and continental keels on plate motion, net rotation of lithosphere, and the geoid. *Journal of Geophysical Research: Solid Earth*, 106(B1):703–712. _eprint: https://onlinelibrary.wiley.com/doi/pdf/10.1029/2000JB900364.

Zhu, D.-C., Li, S.-M., Cawood, P. A., Wang, Q., Zhao, Z.-D., Liu, S.-A., and Wang, L.-Q. (2016). Assembly of the Lhasa and Qiangtang terranes in central Tibet by divergent double subduction. *Lithos*, 245:7–17.
